# Characterization of a CholesteroNitrone (ISQ-201), a Novel Drug Candidate for the Treatment of Ischemic Stroke

**DOI:** 10.3390/antiox9040291

**Published:** 2020-03-31

**Authors:** Emma Martínez-Alonso, Alejandro Escobar-Peso, Maria I. Ayuso, Rafael Gonzalo-Gobernado, Mourad Chioua, Juan J. Montoya, Joan Montaner, Israel Fernández, José Marco-Contelles, Alberto Alcázar

**Affiliations:** 1 Department of Research, IRYCIS, Hospital Ramón y Cajal, Ctra. Colmenar km 9.1, 28034 Madrid, Spain; 2 Neurovascular Research Group, Institute of Biomedicine of Seville, IBiS, Hospital Universitario Virgen del Rocío, Av. Manuel Siurot s/n, 41013 Seville, Spain or; 3 Laboratory of Medicinal Chemistry (IQOG, CSIC), C/Juan de la Cierva 3, 28006 Madrid, Spain; 4 Isquaemia Biotech SL, Scientific Technological Park, C/Astrónoma Cecilia Payne s/n, 14014 Córdoba, Spain; 5 Neurovascular Research Laboratory, Institut de Recerca Vall d’Hebron, Autònoma University of Barcelona, 08035 Barcelona, Spain; 6 Departamento de Química Orgánica I y Centro de Innovación en Química Avanzada (ORFEO-CINQA), Complutense University of Madrid, Av. Complutense s/n, 28040 Madrid, Spain

**Keywords:** antioxidant, brain ischemia, hydroxyl radical, ischemic stroke, neuroprotection, nitrones, steroids, reactive oxygen species

## Abstract

Nitrones have a well-recognized capacity as spin-traps and are considered powerful free radical scavengers, which are two important issues in hypoxia-induced oxidative stress and cell death in brain ischemia. Consequently, nitrones have been proposed as therapeutic agents in acute ischemic stroke (AIS). In this paper, we update the biological and pharmacological characterization of ISQ-201, a previously identified cholesteronitrone hybrid with antioxidant and neuroprotective activity. This study characterizes ISQ-201 as a neuroprotective agent against the hypoxia-induced ischemic injury. Transitory four-vessel occlusion and middle cerebral artery occlusion (tMCAO) were used to induce cerebral ischemia. Functional outcomes were determined using neurofunctional tests. Infarct area, neuronal death, and apoptosis induction were evaluated. In addition, ISQ-201 reactivity towards free radicals was studied in a theoretical model. ISQ-201 significantly decreased the ischemia-induced neuronal death and apoptosis, in a dose-dependent manner, showing its therapeutic effect when administered up until 6 h after post-ischemic reperfusion onset, effects that remained after 3 months from the ischemic episode. Furthermore, ISQ-201 significantly reduced infarct volume, leading to recovery of the motor function in the tMCAO model. Finally, the theoretical study confirmed the reactivity of ISQ-201 towards hydroxyl radicals. The results reported here prompted us to suggest ISQ-201 as a promising candidate for the treatment of AIS.

## 1. Introduction

Strokes represent the second largest cause of death worldwide. The ischemic subtype accounts for approximately 80% of registered cases [1], which is caused by a decrease in cerebral blood flow that affects the whole or part of the brain (global or focal cerebral ischemia, respectively). Reduction of blood flow usually originates from a thrombus or embolus occluding a blood vessel or by systemic hypoperfusion in the brain. As a consequence of the decrease in blood supply, which is modulated by its severity, duration, and area of the brain that is affected, diverse effects are triggered within the components of the neurovascular unit. This set of processes, also known as the ischemic cascade, leads to the loss of cell function and, eventually, to the loss of cell integrity and death [2]. 

In the last decades, a plethora of strategies has been directed against different components of the ischemic cascade to find an adequate approach for the treatment of ischemic disease, overcoming the limitations of recombinant tissue plasminogen activator (rtPA) for thrombolysis, the only approved agent in the treatment of ischemic stroke by the US Food and Drug Administration (FDA). Either alone or in combination with other strategies, antioxidant therapy has long been considered an interesting field of research regarding the relevance of oxidative stress in an ischemic episode [3]. Briefly, during the duration of occlusion, but especially after recanalization, when a massive flow of oxygen reaches the compromised tissue, an overproduction of highly reactive oxygen and nitrogen species (ROS and RNS) takes place [4]. If uncontrolled by the endogenous antioxidant systems, which are compromised due to energy deprivation, radical species might further contribute to exacerbating the damage of the ischemia/reperfusion period. Antioxidant drugs are more often aimed at scavenging radical species (i.e., radical traps), to inhibit the ROS/RNS enzyme producers, or to recover or increase the activity of endogenous antioxidant systems (glutathione, SOD, Nrf2, etc.) [5].

Nitrones were initially developed as detection tools of radical species due to the formation of stable nitrone–radical adducts that can be detected by electron paramagnetic resonance methods [6]. In the following years, after the establishment of the pathological role of free radicals in several diseases, the trapping behavior of nitrone–radicals was suggested to be a relevant feature in the fight against oxidative stress [7]. *N*-*tert*-Butyl-α-phenylnitrone (PBN), which is extensively studied in spin-trapping experiments, was reported to exert a protective effect in rats, after traumatic brain injury [8]. After some development, the PBN-derived nitrone NXY-059 (which has a greater solubility than the parent compound) was described to be a good neuroprotective agent in preclinical models of ischemic stroke. This led to it being considered as a candidate for clinical trials [9,10]. Phase I and II studies revealed adequate pharmacokinetic characteristics and tolerability [11,12,13]. Phase III studies, however, were not able to show a conclusive beneficial effect when compared to placebo, a fact that led to NXY-059 withdrawal from the clinical trial path [14]. 

From this point, and despite the lack of benefit of NXY-059 in clinical phases, nitrone development has remained an appealing field of research, in the search for new candidates for the treatment of ischemic stroke and other diseases. It is the nature, position, and connectivity of the substituents on the nitrone group (the *N*-oxide of an imine), which are the main features modulating the chemical and pharmacological activity of nitrones. Thus, radical trapping activity can be easily modified with subtle chemical changes on the main scaffold, leading to more potent drugs. Nevertheless, this confers upon nitrones a great versatility, which makes it possible to find compounds that are not only able to act as radical traps but which also serve other functions, as examples of pleiotropic drugs.

Drugs aimed for the treatment of ischemic stroke must feature precise characteristics, regarding stroke physiopathology, in order to increase the chances of success. Even in the best situations, patient arrival to the hospital, diagnosis, and start of treatment span several hours since the production of the occlusion. Treatments must, therefore, be effective and safe in an extended time window, covering the period required before treatment and that for recanalization—if not spontaneous.

Recent studies have also pointed out the relevance of long-term recovery in patients who have suffered a stroke. The average age of stroke incidence has dramatically decreased in the last years, mainly because of poorer lifestyles and accumulated risk factors in younger individuals [15]. Apart from the sequelae common to older patients, these young adults (i.e., younger than 50) might eventually develop compromised functional, neuropsychiatric, or cardiovascular outcomes in the long-term yet earlier in their life, conditioning their extended life expectancy [16]. Consequently, the long-term effect should also be a priority in the drug development process and not only during the observation phase after drug approval, i.e., it should also be considered during the preclinical development by taking the limitations of experimental models into account. 

Previously, our group reported the first approach into the biological activity of a cholesterol-derived nitrone (i.e., cholesteronitrone) as a potential treatment for ischemic stroke [17]. ISQ-201, previously named as ChN 2, was identified as a promising candidate after it was administered to primary neuronal cultures subjected to oxygen and glucose deprivation (OGD) as a model of experimental ischemia [17]. Cell viability studies revealed a good neuroprotective effect in the micromolar range, which prompted us to explore the ISQ-201 effect on an in vivo model of cerebral ischemia. Intraperitoneal administration of the candidate at the onset of reperfusion after cerebral ischemia afforded a neuroprotective effect, when compared to the vehicle group [17]. Following these promising results, we decided to continue exploring the biological and pharmacological properties of ISQ-201.

## 2. Materials and Methods

### 2.1. Computational Methods

All calculations reported in this paper were performed with the Gaussian 09 suite of programs [18]. Electron correlation was partially taken into account by using the hybrid function that is usually denoted as B3LYP [19,20,21], in conjunction with the D3 dispersion correction suggested by Grimme et al. [22], using the standard double-ζ quality def2-SVP [23] basis set for all atoms. Geometries were fully optimized in solution without any geometry or symmetry constraints. All species were characterized by frequency calculations [24], and had definite positive Hessian matrices. Frequency calculations were also used to determine the difference between the potential (E) and Gibbs (G) energies, G − E, which contained the zero-point, thermal, and entropy energies. Potential energies were refined in solution, E_sol_, by means of single point (SP) calculations at the same level, with a larger basis set, def2-TZVPP [23], where all elements were described with a triple-ζ + a polarization quality basis set. Solvent effects (solvent = water) were considered in the single-point calculations, using the polarizable continuum method (PCM) [25,26,27]. This level was denoted by PCM (water)-B3LYP-D3/def2-TZVPP// B3LYP-D3/def2-SVP. The ΔG_R_, a given in the text, were obtained from the Gibbs energy in solution, G_sol_, which was calculated by adding the thermochemistry corrections, G − E, to the refined SP energies, E_sol_, i.e., G_sol_ = E_sol_ + G − E.

### 2.2. Synthesis

Cholesteronitrone ISQ-201 was prepared and purified as described in Section A of the Supplementary Material and as reported in [17].

### 2.3. Pharmacokinetics Study

For the study of the variation of the concentration of ISQ-201 in plasma, male Wistar rats (10–12 weeks, Charles River) were randomly divided into three groups (n = 6 per group) and treated intravenously, as a bolus with different concentrations of ISQ-201 (0.07, 0.25, and 1.0 mg/kg, respectively). Plasma samples were taken 0.25, 0.5, 1, 2, 4, and 8 h after the administration and the ISQ-201 concentration was determined by LC–MS/MS analysis. A noncompartmental analysis was performed for the determination of the toxicokinetic parameters, using the Phoenix 64 WinNonlin program (version 8.0). This study was carried out by Swiss BioQuant AG (Reinach, Switzerland).

### 2.4. Animal Model of Global Cerebral Ischemia, Experimental Design, and Treatment 

Transient forebrain ischemia was induced in adult male Wistar rats (10–12 weeks, Charles River) by the standard four-vessel occlusion model (4VO) described previously [28,29]. Briefly, both vertebral arteries were irreversibly occluded by electrocoagulation under anesthesia, with a mixture of atropine, ketamine, and diazepam (0.25, 62.5, and 5 mg/kg, respectively) delivered through intraperitoneal injection. After 24 h, ischemia was induced by carotid occlusion with atraumatic clips, for 15 min, and then the clips were removed from the carotid arteries to allow reperfusion. Body temperature was maintained at 37 °C during the surgical procedure. The animals were studied after 5 days of reperfusion (R5d) after which they were sacrificed. Sham control animals were prepared in the same way, without carotid occlusion. We performed a power analysis (http://www.biomath.info/power/ttest.htm) to determine the sample size. Significance level and statistical power were set at 0.05 and 0.8 (80%), respectively, which afforded a sample size of <6 subjects per group. The treatments were administered with concealed allocation, and in randomized order, according to a computer-generated randomization program. Ischemic animals were treated with a vehicle solution (saline/ethanol, 90:10 vol/vol) or nitrone ISQ-201, dissolved in a vehicle solution through a single intravenous tail injection, when the carotid arteries were unclamped for reperfusion. In therapeutic window experiments, the animals were treated once at the reperfusion onset or at the set reperfusion time points, after ischemia. For the administration, at 6 h and 48 h of reperfusion after ischemia, the animals were anesthetized with isoflurane for 2–3 min and 5 min, respectively, prior to the treatment administration. An independent researcher prepared the treatments for each animal, according to the randomization schedule. Data of each experimental group were normalized to each vehicle control group. All procedures associated with animal experiments were approved by the Animal Care Ethics Committee of the Hospital Universitario Ramón y Cajal (Madrid, Spain, code no. 06/2015) and were performed according to the ARRIVE guidelines.

### 2.5. Evaluation of Neurological Deficits

Neurological deficits in rats subjected to global cerebral ischemia were blindly evaluated after 5 days of reperfusion (R5d). General deficit and deficit in movement and sensory states were quantified, according to a scale ranging from 0 (best) to 10 (rats had a depressed level of consciousness). This scale was validated in an entire cohort of R5d animals (n = 20), as described previously [17].

### 2.6. Behavioral Tests

For the long-term studies, animals were subjected to behavioral tests, in order to assess their spontaneous spatial alternation and spatial memory skills, as markers of neurological damage present in longer periods, after the surgical procedure. According to previous literature [30], we selected the Y maze, in which both the exploration activity (spatial recognition) and spatial memory were evaluated.

For the exploration activity and spatial recognition, control animals and ischemic animals treated with the vehicle or ISQ-201 were placed in the center of the maze and let to explore for 8 min. The total number of arms that the animal entered indistinctly (number of arm entries) and the times that the animal explored consecutively—the three different arms of the maze (number of alternations or triads)—were registered by an observer blind to the treatment.

For the assessment of spatial memory, the control animals and ischemic animals treated with the vehicle or ISQ-201 were placed into one of the arms of the Y maze (start arm) and were allowed to explore the maze with one of the arms closed for 10 min (training trial). After 1 h, the rats were returned to the Y maze by placing them in the start arm and allowing them to freely explore all three arms of the maze for 3 min. The arm of the first choice of entry, the new or old, was registered manually by an observer blind to the treatment.

### 2.7. Brain Sections

After 5 days of reperfusion (R5d), the animals were euthanized by transcardiac perfusion, under deep anesthesia. A washout step with 200 mL of saline via the left ventricle was followed by a fixation with 4% (*w*/*v*) paraformaldehyde solution in PBS. Brains were then removed and post-fixed in the same solution, overnight at 4 °C, sequentially washed with 10%, 20%, and 30% (*w*/*v*) sucrose in PBS, and embedded in Tissue-Tek O.C.T. (Sakura Finetek), before freezing at −80 °C. Brain coronal sections containing the hippocampus were obtained by cryostat sectioning at the level of interaural line +5.7 ± 0.2 mm on real capillary gap microscope slides (Dako).

### 2.8. Neuronal Death Evaluation

Brain cryosections (10 µm thick) from ischemic animals reperfused for 5 days were used after fixation to detect neuronal death by Fluoro-Jade B staining [28], and visualized by fluorescence microscopy. Labeled (dead) neuronal cells (in green) were counted, as in TUNEL assay (see below). Data from different animals of each experimental group were independently analyzed by two observers, and treatment information was kept concealed throughout the study. 

### 2.9. TUNEL Assay

Apoptotic neurons within the brain sections were detected by using the Terminal deoxynucleotidyl transferase-mediated dUTP Nick-End Labeling (TUNEL) assay (Promega). For this test, 5-µm-thick coronal cryostat brain sections containing the dorsal hippocampal formation were obtained, as described above, and post-fixed with 4% formaldehyde in PBS for 5 min, at room temperature. After washing in PBS for three times, the sections were permeabilized with 0.1 M sodium citrate, pH 6.0, for 1 min at 95 °C, then quickly cooled in ice and washed in PBS for 5 min at room temperature. Next, the brain sections were incubated with a blocking solution (0.1 M Tris-HCl, pH 7.5, 3% bovine serum albumin, and 20% fetal bovine serum), washed in PBS for 5 min, and post-fixed with 4% formaldehyde in PBS, for 5 min. After washing in PBS for three times, the sections were incubated with a buffer kit for 10 min, and then incubated with terminal deoxynucleotidyl transferase (TdT) and fluorescein-12-dUTP for 1.5 h at 37 °C, as described by the supplier. The reaction was stopped by extensive washing in saline-sodium citrate buffer (30 mM sodium citrate in 0.3 M NaCl) at room temperature. After washing in PBS for three times, the sections were then mounted with coverslips in an antifade solution, with a glycerol-buffer containing p-phenylenediamine and 30 µM bisbenzimide (Hoechst 33342), for nuclear staining. 

The hippocampal CA1 subfield and cerebral and lateral cortex fields from a given section were analyzed with fluorescence microscopy (40× objective), to count the number of apoptotic nuclei (green). A grid of 330 × 220 µm was used to count the cells in the regions of interest and digitized with a color CCD camera (1280 × 960 pixel resolution). TUNEL-positive cells were counted by two independent observers with a total area of 1.017 mm^2^ analyzed per section. Four sections per brain sample were averaged per experiment and the treatment information was kept concealed throughout the study.

### 2.10. Animal Model of Focal Cerebral Ischemia

Transient occlusion of the distal branch of the middle cerebral artery (tMCAO) was induced in male C57BL/6 mice (9–11 weeks old, Charles River), as described previously [31]. Mice were anesthetized with 4% isoflurane for induction and 1.5–2% isoflurane for maintenance (in 79% N_2_/21% O_2_). After drilling a small hole on the temporal bone at the level of the distal portion of the middle cerebral artery (MCA), the artery was compressed for 60 min, with a 30-G needle by using a micromanipulator. Body temperature was maintained between 36.5–37.5 °C and cerebral blood flow was monitored using laser-Doppler flowmetry to confirm MCA occlusion. Buprenorphine (0.05–0.1 mg/kg) was administered subcutaneously immediately before the procedure. A total of 33 mice were subjected to tMCAO, according to the study design. A power analysis was performed to determine the sample size (significance level set at 0.05, and the power set at 0.8 (80%)), which identified the need for 8 mice to be included in each experimental group. Additional mice were allocated by taking into account mortality and technical issues that arise during the experiments, based on experience. The surgical inclusion criteria were—a reduction in blood flow to <25% of baseline value during the ischemia period, and a recovery of 75% of the baseline value in the reperfusion period. Mice that met the following criteria were excluded—(i) mice that failed to meet the inclusion criteria explained above (4 mice); and (ii) mice that died during the induction of MCA occlusion (2 mice). Mice were randomly assigned by using a randomization software to the following experimental groups (n = 9 per group)—vehicle (saline-ethanol 90:10 vol/vol), ISQ-201 0.05 mg/kg, and ISQ-201 0.1 mg/kg in vehicle. The treatments were administered intraperitoneally at the onset of the reperfusion period, by a blinded investigator. All procedures were approved by the local Animal Care Committee and were conducted in compliance with the ARRIVE guidelines, the Spanish legislations, and EU directives.

### 2.11. Motor-Deficit Evaluation, Grip Strength Test

Neurobehavioral studies were performed to evaluate ischemic outcomes and monitor motor function after focal ischemia. A grip strength test was performed 24 h before the surgery and 24 and 48 h after tMCAO, by a researcher blinded to the experimental conditions. This test was designed to assess the maximum force displayed by mouse forelimbs (in grams), using a metallic grid connected to a force sensor (Bioseb). A total of six trials were conducted for each test and the strength value (expressed in grams) was calculated as the mean of all six trials.

### 2.12. Infarct Volume Evaluation

The size of infarction was evaluated 48 h after tMCAO, using 2,3,5-triphenyltetrazolium chloride (TTC) stain [32]. Mice were sacrificed by transcardiac perfusion with ice-cold saline, under deep anesthesia. Brains were removed and cut into 1-mm-thick coronal sections and stained with 2.5% TTC in saline, at room temperature for 20 min. All analyses were performed in a blinded manner. The infarct areas were analyzed using the Image-J software and the infarct volume was determined through linear integration of the quantified lesion areas and the distances over the sections. To avoid the brain edema effects, the infarct area was corrected by the ratio of the area of the ipsilateral and the contralateral hemisphere.

### 2.13. Statistical Analysis

Data from each treatment and the different animals from each experimental condition or group were independently analyzed and their averaged values were used for statistical analysis. The treatment information was kept concealed throughout the study. Data were represented as mean ± SE. Analysis of variance (ANOVA) or the non-parametric Kruskal–Wallis test (for scoring variables) were performed to compare the data between multiple concentrations or groups, followed by post-hoc tests, to compare pairs of group means when analysis of variance was significant. Statistical significance was set at *p* < 0.05, using Prism statistical software (GraphPad Software).

## 3. Results

### 3.1. In Silico DFT-Study

Based on previous biological data on nitrone ISQ-201 [17] and as superoxide (O_2_^•–^) and hydroxyl (HO^•^) radicals play a major role [33,34] among toxic radical oxygenated species involved in an ischemic event, we investigated the reactivity of these species with nitrone ISQ-201 using density functional theory (DFT) methods, to investigate the exceptional neuroprotective profile of this compound. To this end, and by using a model system of ISQ-201 where the long lateral chain was replaced by an iso-propyl group (ISQ-201’), we explored the thermodynamics of the addition reaction of the oxygen-centered radicals HO^•^ and O_2_^•–^ to ISQ-201’, as well as the most favorable hydrogen abstraction reactions promoted by these species.

**Scheme 1:**
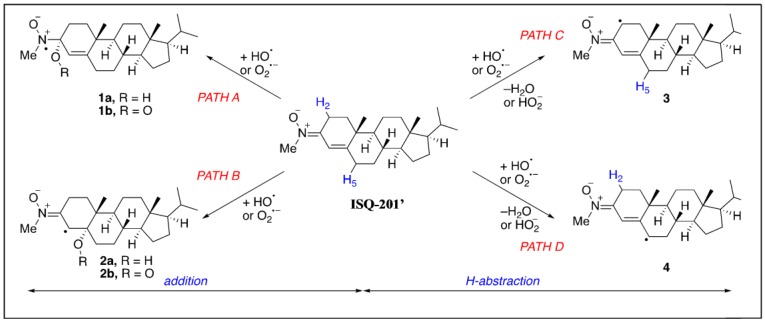
Possible reaction paths for the reaction of nitrone ISQ-201 and oxygen-centered radicals HO^•^ and O_2_^•–^.

Four different processes, shown in Scheme 1, are therefore considered. These include the addition of the oxygen-radicals directly to the nitrone (1,2-addition, path A) and, alternatively, to the conjugated double-bond (i.e., 1,4-Michael type addition, path B), and the hydrogen abstractions involving either H2 (path C) or H5 (path D).

**Figure 1:**
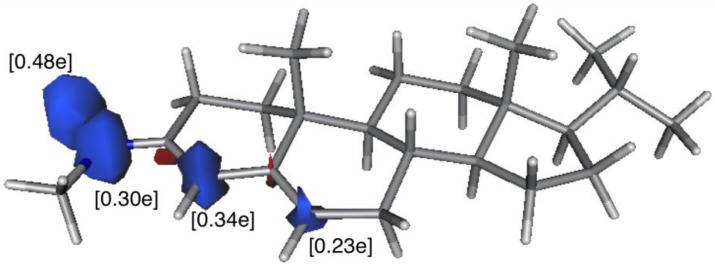
Computed spin density (isovalue = 0.01 au) for compound **4** of Scheme 1.

Table 1 shows the computed free reaction energies in water (∆G_R_, at 298 K) for the above- commented processes. From the data in this Table, it becomes obvious that the reactions involving the superoxide radical are unfeasible, given the high endergonicities computed for these processes. In sharp contrast, all reactions involving the hydroxyl radical are highly exergonic, therefore, suggesting a favorable reaction of nitrone ISQ-201 and this radical. Moreover, the abstraction reaction of H5 (PATH D), which leads to radical species 4 (see Scheme 1) and a molecule of water, becomes the most thermodynamically preferred pathway. The remarkable stability of the latter species can be ascribed to the delocalization of the unpaired electron into the entire conjugated system, which also involves the nitrone moiety. Indeed, the spin density computed for species 4 (see Scheme 1) confirms that the unpaired electron is fully delocalized into this conjugated fragment (Figure 1).

### 3.2. Dose-Response Study

Previous studies of ISQ-201 effective concentration determined an optimal concentration range between 1.0 and 10 µM in primary neuronal cultures subjected to an oxygen and glucose deprivation (OGD) experimental ischemia to achieve significant neuroprotection values [17]. Considering the observed solubility and the penetration profile predicted for ISQ-201, doses of 0.05 and 0.1 mg/kg were administered through intraperitoneal injection at the onset of reperfusion, in animals subjected to cerebral ischemia [17]. As was previously reported, significant decreases in neuronal death and apoptosis, as well as in neurodeficit score, were obtained for both concentrations [17].

**Figure 2:**
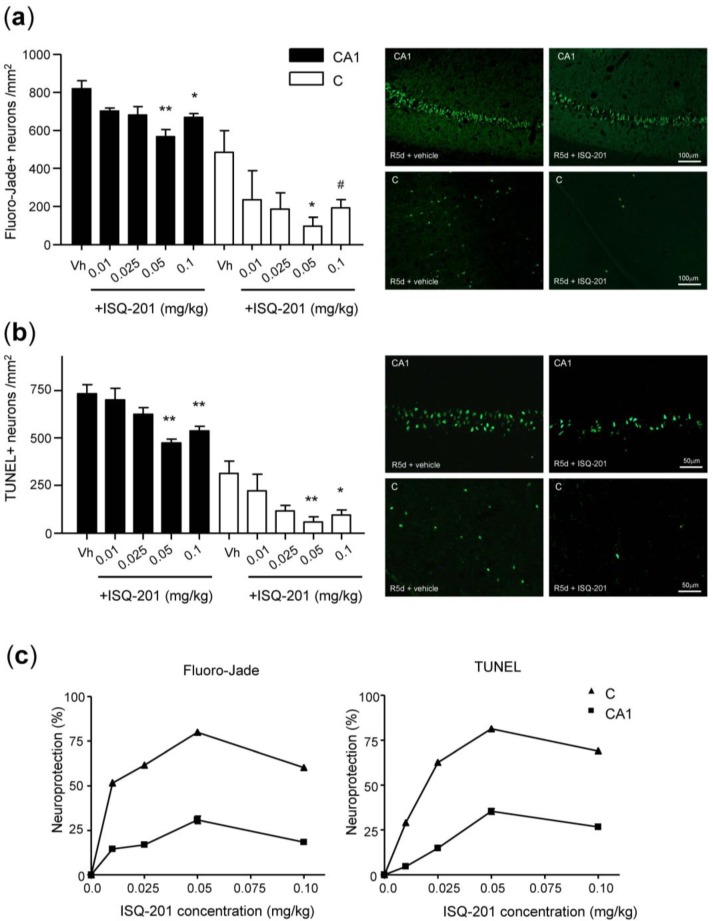
Dose-response curve of ISQ-201. Ischemic animals were treated with a vehicle or ISQ-201 (range 0.01–0.1 mg/kg) by an intravenous injection, at the onset of reperfusion after global cerebral ischemia, and ISQ-201 effect was evaluated by assessing the neuronal death at 5 days of reperfusion (R5d). (**a**) Brain sections from vehicle- and ISQ-201-treated animals (R5d+vehicle and R5d+ISQ-201, respectively), were fixed and stained with Fluoro-Jade B to detect neuronal death. Fluoro-Jade-positive dead neurons were visualized by fluorescence microscopy (in green) in the hippocampal CA1 (CA1) and cortical (C) regions (images), and counted, as described in Materials and Methods (bar graph). (**b**) Brain sections as in (**a**) were used for apoptosis detection by TUNEL assay after fixation, and were visualized by fluorescence microscopy (in green) in the CA1 and C regions (images). TUNEL-positive cells were counted in the CA1 and C regions, as described in the Materials and Methods (bar graph). In (**a**) and (**b**), images are representative results of CA1 or C regions from vehicle- and ISQ-201-treated (0.05 mg/kg) animals. In bar graphs, data represent the mean of 6−11 independent animals per group, and the error bars indicate the SE. * *p* < 0.05 and ** *p* < 0.01, when compared with their respective R5d+vehicle (Vh), through Dunnett’s post-test, and # *p* < 0.05, when compared by Student’s *t*-test, after ANOVA. In (**c**), data from (**a**) and (**b**) are represented as neuroprotective activity of the different concentrations of ISQ-201. Neuroprotection was defined as the percentage achieved from the neuronal death or apoptosis value in the R5d+vehicle (Vh) (0% value), to the absence of neuronal death or apoptosis (100% value). Data are represented as mean ± SE.

In our attempt to improve the applicability of the treatment with ISQ-201, we decided to explore the suitability of an intravenous administration. According to the doses studied previously, we selected a concentration range of 0.01–0.1 mg/kg. Administration of ISQ-201 or a vehicle solution was carried out by an intravenous tail injection at the reperfusion onset, after the global ischemia, according to the procedure described in the Materials and Methods section. Animals were let to recover for 5 days (R5d), after which neuronal death, apoptosis, and the neurodeficit score were assessed. Results are shown in Figure 2.

#### 3.2.1. Analysis of Neuronal Death

Blood supply cessation in the global ischemia model, during a 15-min-induced neuronal death, especially in the hippocampal CA1 and cortical regions of the brain. This neuronal damage could be observed after 5 days of reperfusion through Fluoro-Jade B staining, as a marker of dead neurons. In these experiments, a decrease in the dead neuron numbers could be observed with ISQ-201 treatment in the whole concentration range tested in the hippocampal CA1 region and in the cerebral cortex, when compared to the group treated with a vehicle (Figure 2a). Remarkably, a significant reduction of the dead neuron number observed in the vehicle group could be observed in those groups treated with 0.05 and 0.1 mg/kg of ISQ-201. 

#### 3.2.2. Analysis of Neuronal Apoptosis

Neuronal apoptosis could be observed as a consequence of the 15-min-long global ischemia, when brain slices were subjected to a TUNEL assay, after 5 days of reperfusion, as described in the Materials and Methods section. Cortical and, especially, hippocampal CA1 regions were the most vulnerable to the blood flow impairment in this experimental model, therefore, being adequate for the observation of potential neuroprotective effects. Results shown in Figure 2b describe the reduction in cell apoptosis observed when animals were treated with ISQ-201 (ranging 0.01–0.1 mg/kg), compared to the vehicle group. Consistent with the Fluoro-Jade B experiments, a significant reduction of apoptotic cells is obtained with ISQ-201 at 0.05 and 0.1 mg/kg, in both CA1 and cortical regions.

#### 3.2.3. Dose-Response Curve

Both, a decrease in neuronal death and the apoptosis results are indicative of the neuroprotective effect of ISQ-201 in the experimental model used in this study. In Figure 2c, 0% neuroprotection is assigned to the cell death or apoptosis obtained for the vehicle group through the Fluoro-Jade B or TUNEL assays, respectively, whereas a complete neuroprotection (100%) stands for the complete absence of cell death or apoptosis. At the most potent concentration (0.05 µM), ISQ-201 exerts a maximum neuroprotection (E_max_) of 80% and 31% in neuronal death assays—in the cortex and hippocampal CA1, respectively—and 81% and 35% in TUNEL assays—again in the cortex and CA1, respectively. Considering the dose-response curve, an EC_50_ could be determined, affording a value of 0.015 and 0.025 mg/kg for the graphical representations in cortex and CA1, respectively (Figure 2c). These results point out the use of the concentrations of 0.05 mg/kg of ISQ-201 as the most adequate among the set of doses evaluated, due to its higher value than EC_50_ which is closer to E_max_, in both, the cerebral regions and the efficacy studies tested.

### 3.3. Therapeutic Window

**Figure 3:**
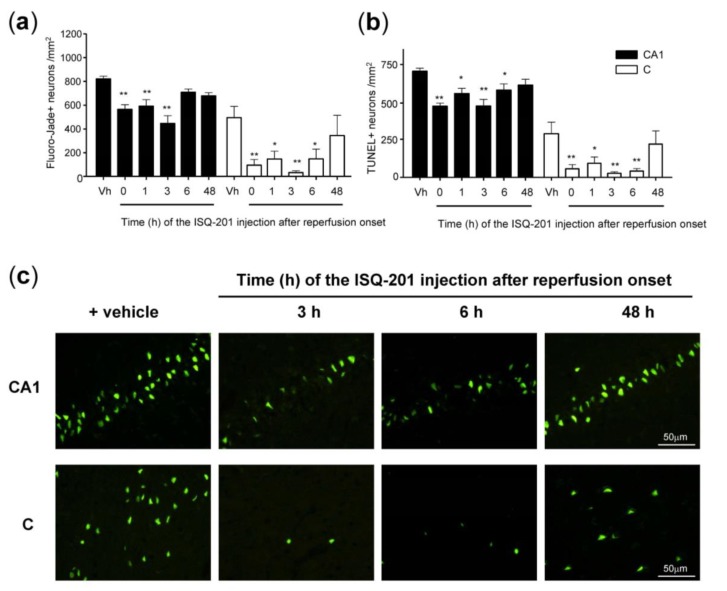
The therapeutic window of ISQ-201. Ischemic animals were treated with vehicle (Vh) or ISQ-201 (0.05 mg/kg), by an intravenous injection at the onset of reperfusion, or at 1, 3, 6, or 48 h of reperfusion after global cerebral ischemia; and the neuroprotection of ISQ-201 was evaluated after 5 days of reperfusion, following ischemia. (**a**) The bar graph represents the neuronal death quantified in brain sections from vehicle- and ISQ-201-treated animals, after fixation and staining with Fluoro-Jade B. Neuroprotection was determined by quantification of the decrease of neuronal death in the hippocampal CA1 (CA1, black bars) and cortical (C, white bars) regions, as described in Materials and Methods. (**b**) The bar graph represents the apoptosis quantified in brain sections from vehicle- and ISQ-201-treated animals, through TUNEL assay. Neuroprotection was determined by quantification of the decrease of neuronal apoptosis in the CA1 and C regions, as described in Materials and Methods. (**c**) Images of the representative results of CA1 or C regions from vehicle- and ISQ-201-treated animals at 3 h, 6 h, or 48 h of reperfusion, after apoptosis detection by TUNEL assay and was visualized by fluorescence microscopy. TUNEL-positive cells were visualized in CA1 and C fields (in green). In (**a**) and (**b**), the data represent the mean of 6−7 independent animals, and the error bars indicate the SE, * *p* < 0.05 and ** *p* < 0.01, compared to their respective vehicle (Vh), through Dunnett’s post-test after ANOVA.

In the following study, delayed administration of ISQ-201, ranging from 0 h to 48 h after reperfusion onset was evaluated in the animal model of cerebral ischemia, to determine its therapeutic window. ISQ-201 was administered as a single intravenous injection of 0.05 mg/kg, the most effective dose was found in the dose-response study (see above). Neuronal death and apoptosis were evaluated after 5 days of reperfusion and a neurodeficit score was determined. In these experiments, each time point of treatment was conducted with a respective vehicle group. No significant differences were found between the vehicle groups (*p* > 0.158, by ANOVA test) and data were represented as normalized, with respect to the vehicle group administered at the onset of the reperfusion (Figure 3a,b).

#### 3.3.1. Analysis of Neuronal Death

As observed with the Fluoro-Jade B staining, the administration of ISQ-201 (0.05 mg/kg) significantly decreased neuronal death at the time points evaluated between 0 and 6 h after reperfusion, when compared to the vehicle group. This effect was observed in both the hippocampal CA1 and the cortical regions. In the cortex, a significant reduction in neuronal death could be observed when ISQ-201 was administered later than 6 h after the reperfusion onset (Figure 3a).

#### 3.3.2. Analysis of Neuronal Apoptosis

Results obtained for the evaluation of neuronal apoptosis were similar to those obtained in the neuronal death analysis. Administration of ISQ-201 at extended periods after reperfusion, significantly reduced apoptosis-positive cells, including at 6 h postreperfusion, both in the hippocampal and the cortical regions, when compared to the vehicle group. Administration at 48 h postreperfusion did not show significant differences in cell apoptosis (Figure 3b). Representative images of TUNEL images are shown in Figure 3c.

#### 3.3.3. Functional Test

**Figure 4:**
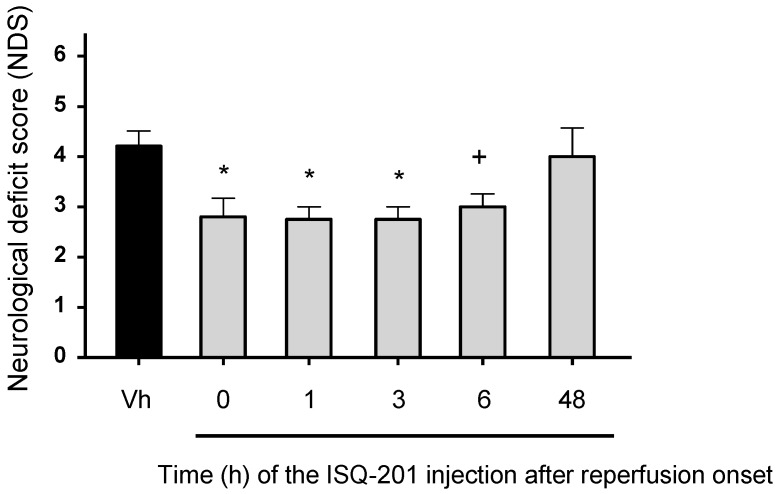
Neurodeficit score outcomes after cerebral ischemia in the therapeutic window study of ISQ-201. The bar graph shows the neurological deficit score (NDS) in ischemic animals at 5 days of reperfusion after ischemia. Animals were treated with the vehicle (Vh) or ISQ-201 (0.05 mg/kg), by an intravenous injection at the onset of reperfusion, or at 1, 3, 6, or 48 h of reperfusion after global cerebral ischemia. The values represent the mean of 6−7 independent animals, and the error bars indicate the SE, * *p* < 0.05 or + *p* < 0.05, compared with the vehicle by Dunn’s or Mann–Whitney post-test, respectively, after the Kruskal−Wallis test.

In order to quantify potential functional impairments, the general state of the animals was assessed. In this test, each variable related to the general state of the animal (breathing, coordination, aspect, etc.) was assigned a grade corresponding to the degree of severity by which that variable was affected. The overall grade accounted for the neurological deficit score (NDS), which represented the general consciousness and neurological activity of the animal (0, healthy animal; 10, the animal had a depressed level of consciousness). As shown in Figure 4, an improvement was observed when the animal was treated with ISQ-201 at any of time points between 0 and 6 h of post-reperfusion, showing significant differences when compared to the vehicle group. When administration occurred after 48 h of reperfusion, ISQ-201 treatment could not improve the neurological state of the animal, compared with the vehicle treatment.

### 3.4. Long-Term Efficacy Assessment

**Figure 5:**
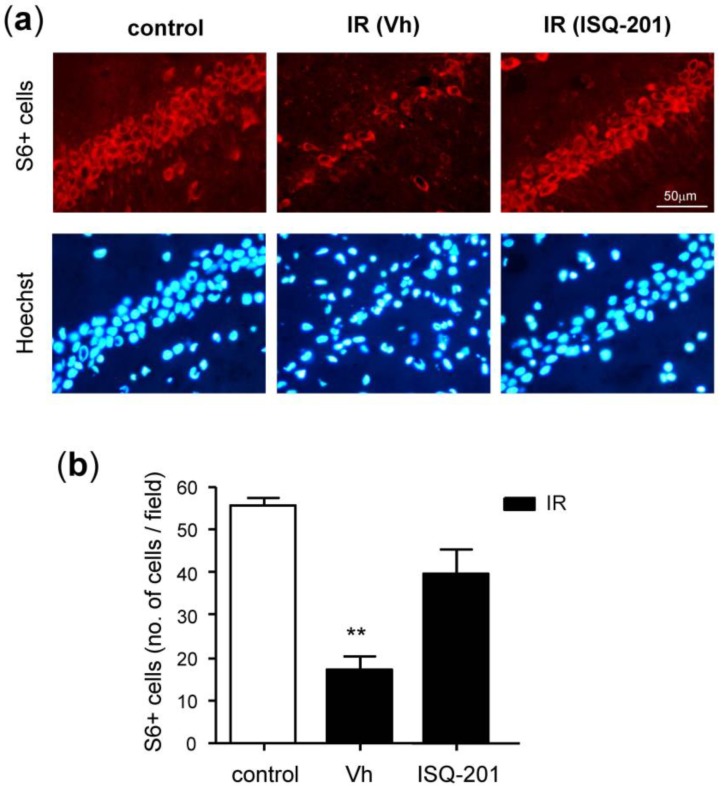
Long-term efficacy study of ISQ-201. (**a**) Brain sections from the sham control animals (control), or from vehicle (Vh)- and ISQ-201-treated (ISQ-201, 0.05 mg/kg) ischemic animals were evaluated for cell detection at 11 weeks after ischemic reperfusion (IR). IR animals were treated by an intravenous injection at the onset of reperfusion, after global cerebral ischemia. Neurons were labelled after fixation with an anti-protein S6 antibody and X red-secondary antibody, were visualized by fluorescence microscopy (in red), and the cells were also stained with the Hoechst dye (in blue). The images are representative results of the hippocampal CA1 region. (**b**) The bar graph represents the number of positive-cells for S6 labeling (S6+ cells), per field of the hippocampal CA1 region from the control animals and the vehicle (Vh)- and ISQ-201-treated ischemic reperfused animals. Values represent the mean of six independent animals, and the error bars indicate the SE, ** *p* < 0.01, compared to the control group, through Dunnett’s post-test, after ANOVA.

As described in the previous sections, a short-term (5 days) neuroprotective effect was achieved when administrating 0.05 mg/kg of ISQ-201 at 0–3 h, after the reperfusion onset. Being aware of the relevance of a long-term effect in the stroke therapeutics, we wanted to assess whether the optimal concentration and administration time had any effect at longer times, after the ischemic episode. In previous experiments, neuronal damage and death produced by the ischemic episode were determined by Fluoro-Jade B and TUNEL assays, 5 days after post-ischemic reperfusion. The study of the effect at longer periods demands a different experimental approach, since the damaged or dead cells are naturally reabsorbed by the brain parenchyma and are not observed through Fluoro-Jade B or TUNEL assays. In the following experiments, we assessed the neuronal death at 11 weeks of reperfusion through immunostaining and quantification of the ribosomal protein S6, a constitutive protein that is present in neuron cytoplasm and is a marker of cell viability [35]. S6 protein staining was observed in the hippocampal CA1 region, one of the most vulnerable brain regions to the ischemic damage. Within this approach, we were able to observe whole neurons, indicating proper cell viability in the brain sections of sham control animals (Figure 5a, control, S6-positive (S6+) cells). Ischemic animals were treated with vehicle or ISQ-201, through intravenous injections, at the onset of reperfusion, after cerebral ischemia and neuronal loss was evaluated in the brain sections, at 11 weeks after treatment (Figure 5a, IR, S6+ cells). Additionally, animal behavior was also evaluated for the neurofunctional outcome assessment.

#### 3.4.1. Analysis of Neuronal Viability

Viable neurons in the hippocampal CA1 region, at 11 weeks after reperfusion were stained with the S6 protein in ISQ-201-treated animals and were compared with vehicle-treated animals (Figure 5a, IR, S6+ cells). In the pictures, a severe reduction in the S6-positive cells could be observed in vehicle-treated animals (Figure 5a, IR Vh). These results showed how 15 min of cerebral ischemia, followed by reperfusion, could devastate and extinguish neuronal cells, in the CA1 region of the brain hippocampus, the region where the short-term memory and spatial memory reside.

Remarkably, it can be observed in the pictures that neurons in the CA1 region from the ISQ-201-treated animals were similar in number and morphology to hippocampal neurons from control animals (Figure 5a, control and IR ISQ-201). Quantification of S6 staining results was carried out by counting S6-positive neurons (viable neurons) in the CA1 region, neurons that were preserved with the administration of ISQ-201 (Figure 5b).

#### 3.4.2. Behavioral Tests

As for neuronal viability studies, previously described functional tests were not adequate for a long-term outcome assessment. Animals might overcome their initial functional deficits due to the nervous system’s inherent plasticity, recovering completely, several days after the ischemia. For this reason, exploration tests assessing spatial recognition, memory, orientation, and curiosity, whose deficits are maintained over a longer period after the insult were needed. In these experiments, we carried out two different behavioral tests, assessing the animal’s spatial recognition and spatial memory, to evaluate long-term cognitive impairment (see the Materials and Methods section for detailed procedure information).

##### Exploration Activity and Spatial Recognition Test

**Figure 6:**
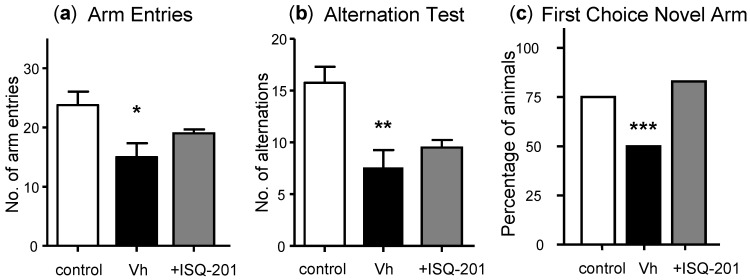
Behavioral tests after cerebral ischemia at long-term. The bar graphs represent the results obtained after three spatial recognition and spatial memory test, to assess the long-term cognitive impairment of control animals and vehicle (Vh)- and the ISQ-201-treated ischemic animals, at 11 weeks after ischemic reperfusion. The values represent the mean of 6 independent tests, performed on different animals. In (**a**) and (**b**), error bars indicate the SE, * *p* < 0.05 and ** *p* < 0.01, as compared to the control group using Dunn’s post-test after Kruskal−Wallis test; in (**c**), *** *p* < 0.001, and as compared to the vehicle using Fisher’s test.

For the exploration activity test, vehicle- or ISQ-201-treated animals were compared to the control group. It was observed that the vehicle group had a significantly higher neurological deficit, compared to the control group (Figure 6a). In contrast, the animals treated with ISQ-201 showed no significant differences with the control group. In the same test, the number of alternations or times that the animals completed a triad (i.e., they explored the three different arms of the maze consecutively) was also assessed as a parameter indicative of their spatial recognition (Figure 6b). As for the assessment of the number of arm entries, the vehicle group showed a significantly higher neurological deficit, when compared to the control, as indicated by the smaller number of triads completed. The ISQ-201-treated group ameliorated their deficit, resulting in no significant differences, when compared to the control.

##### Spatial Memory Test

To evaluate the animals’ long-term spatial memory, they were subjected to a novel arm recognition test in the Y-maze. After the training period, with one of the arms closed, the animals were introduced to the maze again, with two arms available for exploration. The exploration of the new arm as a first choice was indicative of proper spatial memory and, therefore, it was assessed in this test for control, vehicle-, or ISQ-201-treated animals. The results (Figure 6c) showed a decreased percentage of animals within the vehicle group, exploring the new arm as a first choice and indicating an impaired spatial memory. This was ameliorated with the ISQ-201-treatment, which allowed the recovery of values similar to the control group.

### 3.5. Pharmacokinetic Study

**Figure 7:**
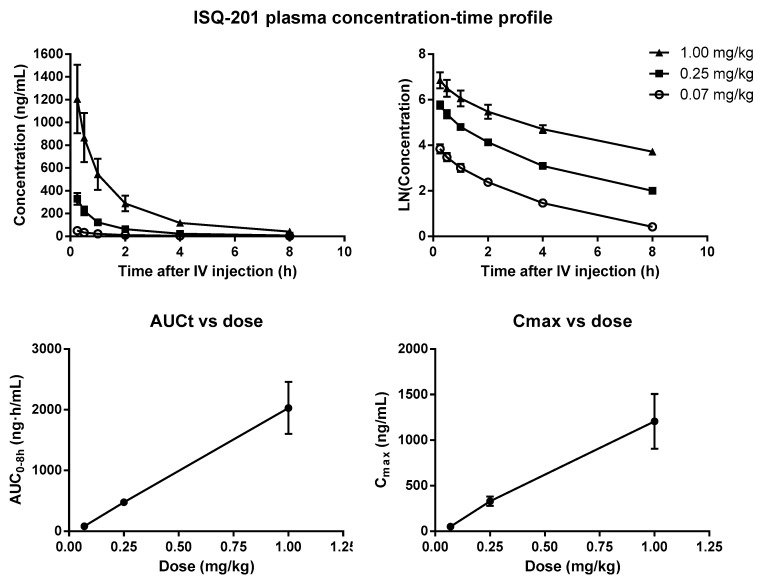
ISQ-201 plasma concentration-time profile after intravenous administration (linear and semi-logarithmic scale in the left and right upper graphs, respectively); and values of AUC_0-8h_ (AUC_t_) and C_max_ versus dose. Data represented as mean ± SE of 6 independent animals.

Study of the variation of ISQ-201 concentration after a single bolus intravenous administration was carried out with different doses of ISQ-201 (0.07, 0.25, and 1.00 mg/kg). Plasma samples were collected 0.25, 0.5, 1, 2, 4, and 8 h after administration, and the ISQ-201 concentration was determined through LC/MS–MS (Figure 7). Pharmacokinetic parameters were calculated through a non-compartmental analysis (Table 2).

ISQ-201 was rapidly detected in the blood, after intravenous administration, and their plasmatic concentration gradually decreased over time. Concentration values over the LLOQ (1.0 ng/mL) were detected even after 8 h (Table 2, C_last_). The half-life around 1.5–2 h showed that ISQ-201 was eliminated quickly. The mean C_max_ and AUC_0-8h_ values of ISQ-201 were linearly related to the dose in the whole studied range (0.07–1.00 mg/kg), indicating that exposure to the drug increased proportionally to the injected dose (Figure 7).

### 3.6. ISQ-201 Treatment in Transient Focal Cerebral Ischemia

**Figure 8:**
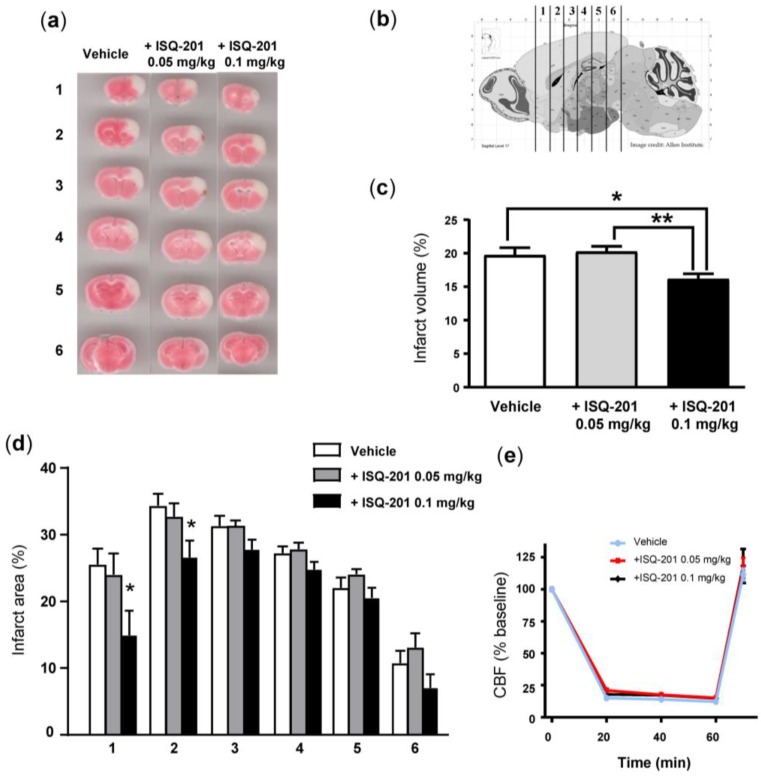
Neuroprotective effect of ISQ-201 in transient focal cerebral ischemia. ISQ-201 treatment reduces infarct size after transient middle cerebral artery occlusion (tMCAO). Vehicle or ISQ-201 (0.05 and 0.1 mg/kg) was administered at the onset of the reperfusion after tMCAO, and infarct size was quantified at 48 h of reperfusion. Brain sections from ischemic animals were stained with 2,3,5-triphenyltetrazolium chloride (TTC) to determine the infarct size. (**a**) Representative images of TTC-stained brain sections of ischemic mice. Six coronal sections (1 mm) of the rostrocaudal axis are shown. (**b**) The schematic figure shows the sections of the coronal slices in the whole brain (illustration from Franklin, K.; Paxinos, G. The Mouse Brain in Stereotaxic Coordinates, 3rd ed.; Academic Press: San Diego, CA, USA, 2007. Image credit: Allen Institute). The bar graphs in (**c**) and (**d**) showed the quantification of the infarct volume and infarct area, respectively, in ISQ-201-treated animals, compared to the vehicle. Values represent the mean of 9 independent animals, and SE is indicated by the error bars, * *p* < 0.05, compared to the vehicle, and ** *p* < 0.01 through Bonferroni’s post-test after ANOVA. (**e**) The cerebral blood flow (CBF) of all studied animals is represented to show that the surgical procedure of tMCAO was properly performed in the three groups. Data are represented as mean ± SE.

**Figure 9:**
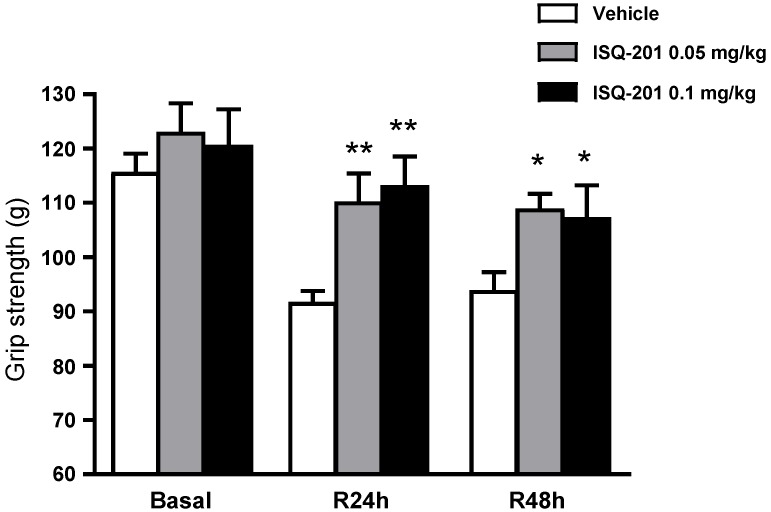
Improvement of the functional outcome of ISQ-201 treatment after transient focal ischemia. The functional motor deficit was evaluated by a grip strength test. Vehicle or ISQ-201 (0.05 and 0.1 mg/kg) was administered at the onset of the reperfusion after tMCAO, and the grip strength values were recorded at 24 and 48 h of reperfusion (R24h and R48h, respectively). The bar graph shows the values corresponding to presurgery animals (basal) and the improved grip strength values in ISQ-201-treated animals at R24h and R48h. Values represent the mean of 9 independent animals, and SE is indicated by error bars, * *p* < 0.05 and ** *p* < 0.01, compared to the vehicle using Bonferroni’s post-test after two-way ANOVA.

To corroborate the neuroprotective effect of ISQ-201 on ischemic stroke, we induced a transient middle cerebral artery occlusion (tMCAO) and evaluated infarct size at 48 h of reperfusion after tMCAO in mice treated with an intraperitoneal injection of ISQ-201. Additionally, motor deficit through a grip strength test was evaluated at 24 h and 48 h after the administration of ISQ-201. Brain sections were stained with TTC and the injured areas were quantified to determine the infarct volume. Furthermore, we tested two doses of ISQ-201 (0.05 mg/kg and 0.1 mg/kg) to perform a dose-response study. Our results showed that the size of the ischemic lesion was reduced in the ISQ-201-treated animals, which was significantly lower in the treated animals at 0.1 mg/kg ISQ-201 dose and with a 21% the reduction in lesion size, with respect to the vehicle-treated group (Figure 8). The analysis of functional outcomes confirmed an improvement in forelimb muscular strength in the ISQ-201-treated animals. Animals treated with ISQ-201 at 0.05 mg/kg or 0.1 mg/kg showed a significant decrease in motor deficit at 24 and 48 h, after treatment, compared with vehicle-treated animals (Figure 9). However, treatment with ISQ-201 at 0.05 mg/kg did not change the infarct area in the brain. The size of the ischemic lesion stained with TTC and the motor function test did not evaluate the same phenomena. TTC staining assessed the ischemic lesion extension and cell death, while the motor function evaluated the neuronal function. Thus, ISQ-201 at 0.05 mg/kg might be unable to reduce the infarct size and cell death, but it could induce recovery and protect resilient neurons in the ischemic penumbra area. In summary, the ISQ-201 treatment reduced the size of infarction and ameliorated the functional motor deficit in mice subjected to tMCAO. These results reinforced that ISQ-201 could be a potential neuroprotective compound against cerebral ischemia damage and could be a promising agent for the treatment of ischemic stroke.

## 4. Discussion

Currently, recanalization strategies, either pharmacological (e.g., rtPA) or mechanical (thrombectomy), were the only therapies approved by the FDA or European Medicines Agency (EMA), for the treatment of acute ischemic stroke (AIS). A substance or therapeutic strategy that is able to minimize the damage produced after ischemia-reperfusion injury is, therefore, highly necessary for use as a single therapy or in combination with the previously mentioned recanalization approaches. To date, despite extensive research in the area, no substance has been found to be effective in improving patient outcomes or decreasing stroke-associated mortality.

Previously, we reported a nitrone compound, ISQ-201—referred to as ChN 2—which was able to exert a neuroprotective effect over the primary neuronal cultures subjected to oxygen and glucose deprivation [17]. Additionally, we reported preliminary results regarding the ISQ-201 effect on lipid peroxidation and ROS levels, which were both decreased [17]. Intraperitoneal administration of ISQ-201 at the onset of reperfusion after global cerebral ischemia showed decreased neuronal death and apoptosis in those regions that were more vulnerable to the ischemia-reperfusion period—i.e., hippocampus and cortex [17]. Regarding these findings, ISQ-201 was upgraded as a lead in the treatment of AIS.

In this paper, we have updated the biological and pharmacological characterization of ISQ-201 as a preclinical candidate for AIS treatment. First of all, the strong and efficient effect predicted by DFT analysis of the presumed favored reactivities of nitrone ISQ-201 with oxygen-centered radical HO^•^ confirmed the interest in this candidate. The hydroxyl radical was the most reactive of the free radicals and are highly harmful in ischemia-reperfusion injuries that follow reoxygenation [36]. Trapping of radical species would decrease oxidative-stress-mediated damage over the cell structures and functionalities that compromise the tissue viability and, therefore, is a desired feature of a compound aimed for the neuroprotection in ischemic stroke. Moreover, we have evaluated the efficacy of different doses of ISQ-201 (0.01–0.1 mg/kg) administered by intravenous injection in an in vivo model of cerebral ischemia. This study identified 0.05 mg/kg as the most effective dose in decreasing neuronal death and apoptosis after 5 days of reperfusion, but also showed an effect on a 0.1 mg/kg dose, although it was not that significant. This lower dose would decrease the probability of adverse effects due to off-target activity, as well as reduce toxicity concerns.

The acute character of stroke diseases make the period between a stroke onset (i.e., when symptoms show up) and the administration of the treatment, a crucial concern in the development of potential therapies and strategies of the health systems. Often, several hours pass between the patient developing the first symptoms and being diagnosed and treated. Additionally, patients might require a recanalization treatment, if not produced spontaneously, which might also determine the effect of additional drugs used. Therefore, the study of a potential treatment must be explored in preclinical models of ischemia in a delayed administration basis, to properly assess its safety concerns, therapeutic effects, and suitability for the clinic.

Considering the relevance of a delayed administration schedule in common clinical practice, we also explored the efficiency of a single ISQ-201 administration in a time window ranging from 0 to 48 h after reperfusion onset. Results reported herein confirm the effectivity of ISQ-201 in decreasing neuronal death, neuronal apoptosis, and general neurofunctional deficits in our preclinical model, even when administered 6 h after stroke. Even though the compatibility of these results with recanalization strategies is still unknown, and correlation with the administration time points in human patients must still be assessed, the extended therapeutic window of ISQ-201 is of paramount relevance and could be decisive in its further development. Nevertheless, whether an antioxidant activity—which is theoretically most effective in the first moments after the recanalization—is the cause of the efficiency found at such long periods after the reperfusion onset, must be explored in further studies.

Increasing life expectancy and earlier incidence of stroke episodes make the assessment of outcomes at longer periods a necessity [15,16], both in clinical and preclinical phases. Being conscious of the obvious limitations, and trying to compare life spans between different species, we explored a 3-month-long post-treatment period in adult rats, which would correspond to a 5–7 years life span in humans [37]. Regardless of the exact equivalence between the two species, this time allows the observation of the evaluation parameters once the brain remodeling action after the injury has been completed or, in other words, when the endogenous neurorepair processes have already finished [38]. Therefore, the biochemical analysis and neurofunctional tests to perform must be able to assess the permanent sequelae that could have resulted from the ischemic episode. The immunofluorescence detection of S6 protein after 11 weeks (3 months) of reperfusion has allowed the observation of the anatomical disruption produced in the hippocampal CA1 region resulting from the ischemic injury. Behavioral tests confirmed this by detecting an impaired performance in the spontaneous spatial recognition and spatial memory tests in those untreated ischemic animals. Remarkably, the ISQ-201 treatment not only avoided short-term deleterious effects, as seen previously in the TUNEL and Fluoro-Jade B experiments at 5 days of reperfusion, but also decreased the long-term functional impairment at a 3-month-long period.

The preclinical pharmacokinetic study played an essential role in the drug development process, yielding important information regarding the compound’s characteristics, quality, mechanism of action, and toxicity. Moreover, it could provide a valuable reference for the design and optimization of clinical trials. In this paper, we reported a preliminary study of the compound concentration variation in plasma for 8 h, after intravenous injection. The results obtained confirmed a linear dose-dependent pharmacokinetics in the dose range studied (1×, 5×, and 20× therapeutic dose), as shown by the C_max_/dose and the AUC/dose plots. Kinetic parameters defined by the clearance and half-life showed an adequate elimination rate. Thus, the half-life of ISQ-201—about 2 h—could be adequate to act as a radical trap within the critical period of ischemic damage. In the ischemic injury, during the occlusion, and mainly after the reperfusion, the overproduction of highly reactive oxygen and nitrogen species take place during the first hours. Hence, the effect induced by ISQ-201 during the first two hours could be decisive enough to avoid irreversible neuronal damage, allowing neuronal repair and survival (i.e., neuroprotection). This neuroprotection would lead to the preservation of the neuronal layers, as observed in long-term experiments.

Furthermore, we decided to assay ISQ-201 in another species and experimental model of cerebral ischemia, a transient focal ischemia model. ISQ-201 decreased the functional motor deficit, and notably produced a reduction in the infarct size in the animals subjected to transient focal ischemia.

## 5. Conclusions

In conclusion, we have reported an update into the ISQ-201 biological activities as a compound for the treatment of ischemic stroke. Furthermore, we have reported some preliminary results into its pharmacological characterization, including pharmacokinetic properties. New compounds to be launched into clinical trials for the treatment of ischemic stroke require an extensive characterization during the preclinical phases addressing the key issues for the effectiveness and safety of the compound. Our results with ISQ-201 invite a continuation of the preclinical studies with this potential therapeutic agent.

## 6. Patents

Marco, José L., and Alcázar, Alberto. Steroidal Nitrones for the Treatment and Prevention of Cerebral Stroke or Ischaemia, Alzheimer and Parkinson Disease, and Amyotrophic Lateral Sclerosis. U.S. Patent 10071106.

## Figures and Tables

**Table 1:** Computed free reaction energies (∆G_R_, at 298 K, in kcal/mol) for the reactions involving ISQ-201’ and radicals HO^•^ and O_2_^•–^. All data were computed at the polarizable continuum method (PCM)(water)–B3LYP-D3/def2-TZVPP//B3LYP-D3/def2-SVP level.

Reaction Path	HO^•^	O_2_^•−^
PATH A	−36.5	+14.5
PATH B	−37.1	+6.9
PATH C	−41.1	+15.6
PATH D	−48.7	+18.5

**Table 2:** Pharmacokinetic parameters of ISQ-201.

Dose (mg/kg)	C_0_^1^ (ng/mL)	C_max_ (ng/mL)	AUC_0–8h_ (ng·h/mL)	C_last_ (ng/mL)	V_d_ (mL)	k_el_ (h^−1^)	Cl (mL/h)	t_1/2_ (h)
0.07	74.6 ± 12.2	50.6 ± 7.4	81.1 ± 9.5	1.53 ± 0.09	364 ± 110	0.317 ± 0.021	105.0 ± 20.5	1.66 ± 0.10
0.25	490 ± 80.2	329.8 ± 51.2	477 ± 42	7.49 ± 0.75	166 ± 27	0.343 ± 0.025	55.4 ± 6.3	1.51 ± 0.12
1.00	1686 ± 424	1206.2 ± 299.5	2030 ± 429	42.03 ± 4.42	300 ± 103	0.286 ± 0.064	53.7 ± 6.1	2.20 ± 0.53

^1^ C_0_: initial concentration; C_max_, maximal concentration; AUC_0-8h_: Area under the curve for the period 0–8 h; V_d_: volume of distribution; K_el_: terminal elimination constant; Cl, terminal clearance; t_1/2_, half-life. Data as mean ± SE of 6 independent animals.

## Supplementary Materials

The following are available online at https://www.mdpi.com/2076-3921/9/4/291/s1, A. Synthesis of Cholesteronitrones ISQ-201/ISQ-202: 1. General methods for chemistry synthesis, 2. General procedure for cholesteronitrone (ChN) synthesis (including spectroscopic data), Figure S1: Synthesis of ChNs E-(ISQ-201), and Z-(ISQ-202) from 5-cholesten-3-one and 4-cholesten-3-one, Figure S2: (A) ^1^H NMR spectrum of ChN ISQ-201. (B) Selective ^1^H NMR nOe spectrum of ChN ISQ-201, and Figure S3: (A) ^1^H NMR spectrum of ChN ISQ-202. (B) Selective ^1^H NMR nOe spectrum of ChN ISQ-202; and B. Synthesis of cholesteronitrone ISQ-201 from natural cholesterol-plant: 1. Oxidation of cholesterol, 2. Isomerization of compound MC880F1, and 3. Synthesis of ISQ-201 plant, including spectroscopic data.
