# Supplementary material for: Characterization of a CholesteroNitrone (ISQ-201), a Novel Drug Candidate for the Treatment of Ischemic Stroke
Source: Antioxidants (Basel). 2020 Mar 31;9(4):291. doi: 10.3390/antiox9040291 (PMC7222207; doi:10.3390/antiox9040291)

Supplementary Material

Characterization of a CholesteroNitrone (ISQ-201), a Novel Drug Candidate for the Treatment of Ischemic Stroke

Emma Martínez-Alonso [1], Alejandro Escobar-Peso [1], Maria I. Ayuso [2], Rafael Gonzalo-Gobernado [2], Mourad Chioua [3], Juan J. Montoya [4], Joan Montaner [2,5], Israel Fernández [6,*], José Marco-Contelles [3], and Alberto Alcázar [1,*]

[1] Department of Research, IRYCIS, Hospital Ramón y Cajal, Ctra. Colmenar km 9.1, Madrid 28034, Spain; emma.martinez@hrc.es (E.M.-A.), alejandro.escobar@hrc.es (A.E.-P.), alberto.alcazar@hrc.es (A.A.)

[2] Neurovascular Research Group, Institute of Biomedicine of Seville, IBiS, Hospital Universitario Virgen del Rocío, Av. Manuel Siurot s/n, Seville 41013, Spain; mayuso-ibis@us.es (M.I.A.), rgonzalo-ibis@us.es (R.G.-G.), jmontaner-ibis@us.es (J.M.)

[3] Laboratory of Medicinal Chemistry (IQOG, CSIC), C/Juan de la Cierva 3, Madrid 28006, Spain; mchioua@gmail.com (M.C.), jlmarco@iqog.csic.es (J.M.-C.)

[4] Isquaemia Biotech SL, Scientific Technological Park, C/Astrónoma Cecilia Payne s/n, Córdoba 14014, Spain; jjmontoya@canaanrd.com

[5] Neurovascular Research Laboratory, Institut de Recerca Vall d'Hebron, Autònoma University of Barcelona, Barcelona 08035, Spain; joan.montaner@vhir.org

[6] Departamento de Química Orgánica I y Centro de Innovación en Química Avanzada (ORFEO-CINQA), Complutense University of Madrid, Av. Complutense s/n, Madrid 28040 Spain; israel@quim.ucm.es

Contents

A. Synthesis of cholesteronitrones ISQ-201/ ISQ-202……………………………………………S2-S9

B. Synthesis of cholesteronitrones ISQ-201 /ISQ-202 from natural cholesterol-plant…………S10-S15

A. Synthesis of cholesteronitrones ISQ-201 /ISQ-202

1. General methods for chemistry synthesis

Reactions were monitored by TLC using precoated silica gel aluminium plates containing a fluorescent indicator (Merck, 5539). Detection was done by UV (254 nm) followed by charring with sulfuric-acetic acid spray, 1% aqueous potassium permanganate solution or 0.5% phosphomolybdic acid in 95% EtOH. Anhydrous Na_2SO_4 was used to dry organic solutions during work-ups and the removal of solvents was carried out under vacuum with a rotary evaporator. Flash column chromatography was performed using silica gel 60 (230-400 mesh, Merck). Melting points were determined on a Kofler block and are uncorrected. IR spectra were obtained on a Perkin-Elmer Spectrum One spectrophotometer. ^{1}H NMR spectra were recorded with a Varian VXR-200S spectrometer, using tetramethylsilane as internal standard and ^{13}C NMR spectra were recorded with a Bruker WP-200-SY. All the assignments for protons and carbons were in agreement with 2D COSY, HSQC, HMBC, and 1D NOESY spectra. The purity of compounds was checked by elemental analyses, conducted on a Carlo Erba EA 1108 apparatus, and confirmed to be ≥ 95%.

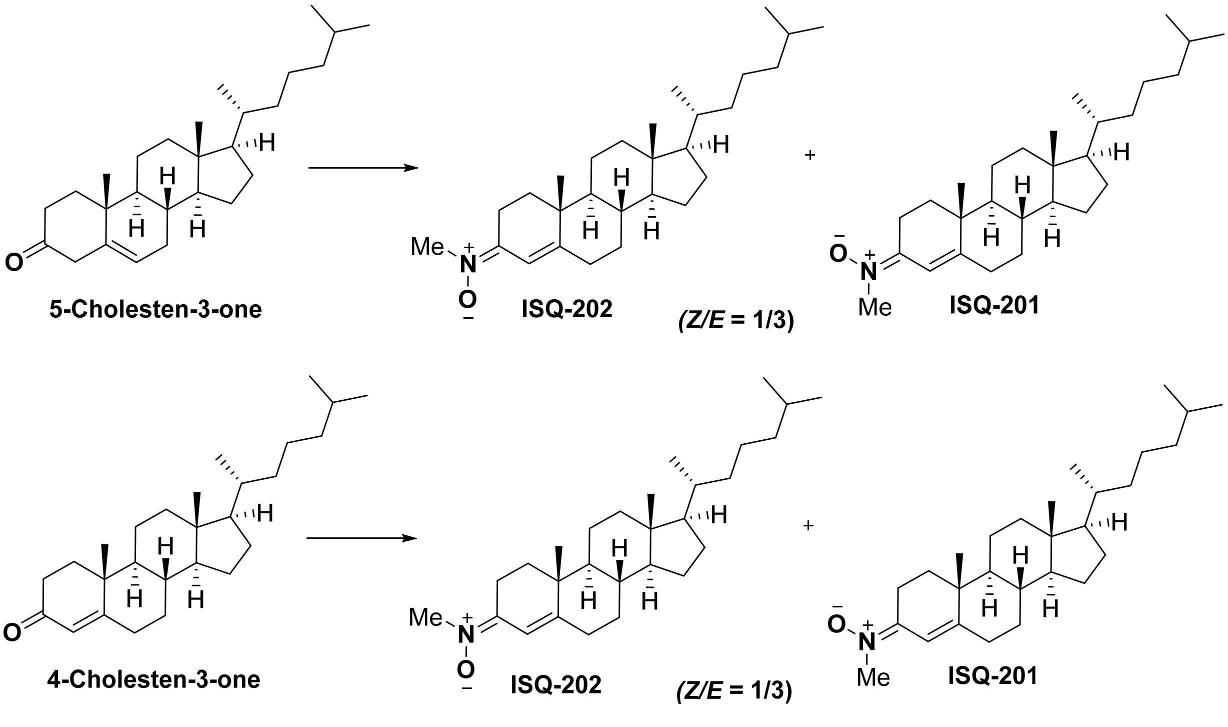

Figure 1. Synthesis of ChNs *E*-(**ISQ-201**), and *Z*-(**ISQ-202**) from 5-cholesten-3-one and 4-cholesten-3-one.

2. General procedure for cholesteronitrone (ChN) synthesis

A solution of the ketone (1 mmol), dry Na_2SO_4 (3 mmol), and triethylamine (2 mmol) were suspended in EtOH. Then, the hydroxylamine hydrochloride (1.5 mmol) was added. The mixture was stirred for 30 s and then exposed to MWI (250 W) at 90 °C during the time indicated for each compound. When the reaction was over (tlc analysis), the solvent was removed under reduced pressure and diluted with water, extracted with AcOEt, dried over anhydrous sodium sulfate, filtered, and the solvent was evaporated. The resultant solid was purified by column chromatography (CH_2Cl_2/MeOH, from 1% to 3%) to give pure compounds *E*-(**ISQ-201**), and *Z*-(**ISQ-202**)[1] (Figure 1).

2.2. Synthesis of ChNs *E*-(ISQ-201), and *Z*-(ISQ-202) from 5-cholesten-3-one. Following the general procedure, reaction of commercial 5-cholesten-3-one (385 mg, 1 mmol), Na_2SO_4 (426 mg, 3 mmol), triethylamine (0.14 ml, 2 mmol), and methylhydroxylamine hydrochloride (126 mg, 1.5 mmol) in ethanol (10 mL), after 7 h, and column chromatography (CH_2Cl_2/MeOH, from 1% to 3%), gave a mixture of **ISQ-201** and **ISQ-202** in a 3:1 ratio, respectively, that after careful chromatography allowed us to obtain pure samples of **ISQ-201** (296 mg, 71%), **ISQ-201 +ISQ-202** (12 mg, 3%), and **ISQ-202** (98 mg, 24%).

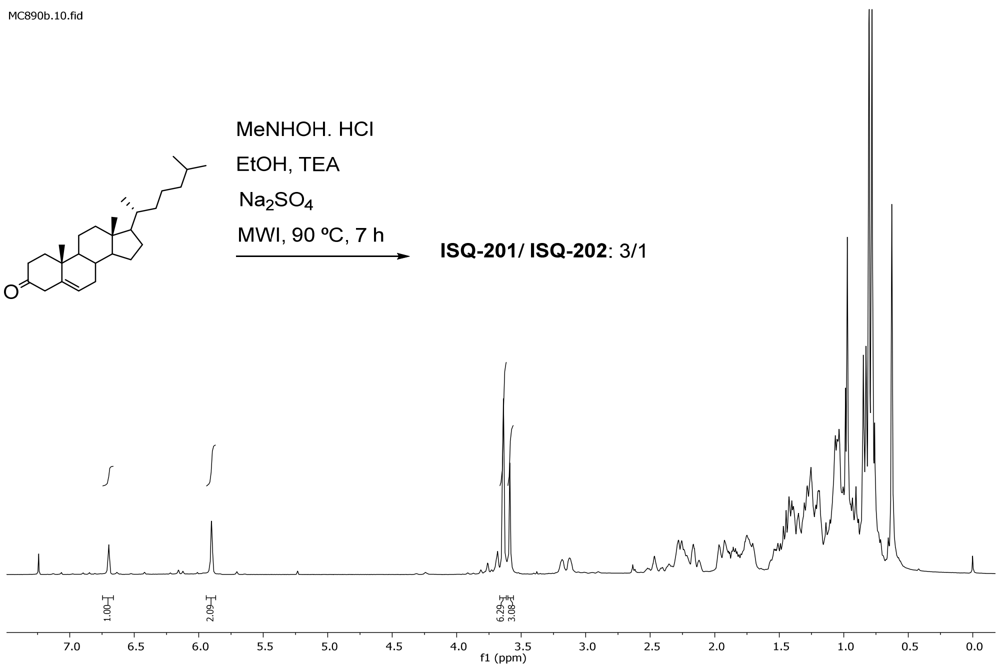

^{1}H NMR spectrum of the crude reaction mixture MC890-TEA

2.3. Synthesis of ChNs *E*-(ISQ-201), and *Z*-(ISQ-202) from 4-cholesten-3-one.

(A) Following the general procedure, reaction of commercial 4-cholesten-3-one (385 mg, 1 mmol), Na_2SO_4 (426 mg, 3 mmol), **triethylamine** (0.14 ml, 2 mmol), and methylhydroxylamine hydrochloride (126 mg, 1.5 mmol) in Ethanol (10 mL), after 12 h, gave a mixture of **ISQ-201** and **ISQ-202**, in a 3:1 ratio, that after careful chromatography (CH_2Cl_2/MeOH, from 1% to 3%), gave **ISQ-201** (273 mg, 66%), **ISQ-201 +ISQ-202** (12 mg, 3%), and **ISQ-202** (91 mg, 22%).

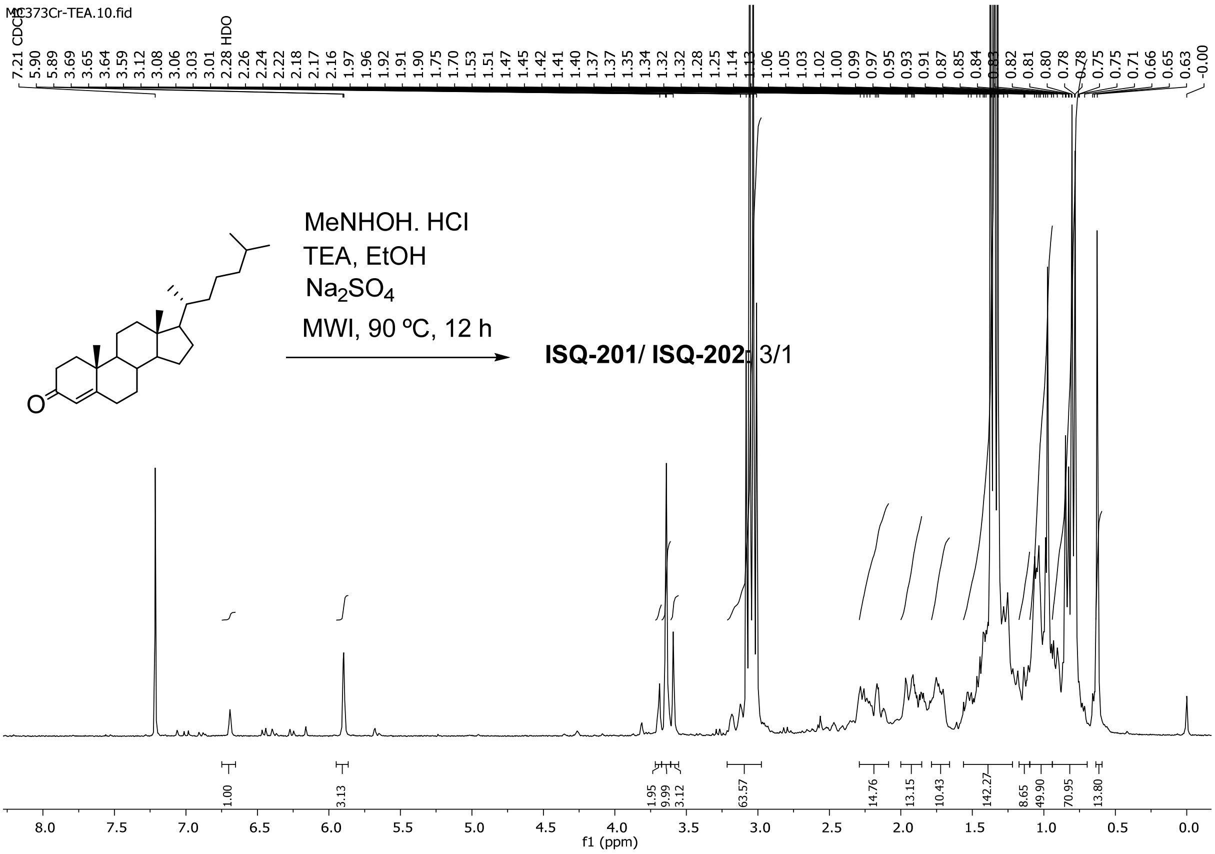

^{1}H NMR spectrum of the crude reaction mixture MC373-TEA

(B) Following the general procedure, reaction of commercial 4-cholesten-3-one (385 mg, 1 mmol), Na_2SO_4 (426 mg, 3 mmol), **$NaHCO_3$** (168 mg, 2 mmol), and methylhydroxylamine hydrochloride (126 mg, 1.5 mmol) in ethanol (10 mL), after 7 h, gave a mixture of **ISQ-201** and **ISQ-202**, in a in a 2:1 ratio, that after careful chromatography (CH_2Cl_2/MeOH, from 1% to 3%), gave **ISQ-201** (205 mg, 49%) and **ISQ-202** (100 mg, 25%).

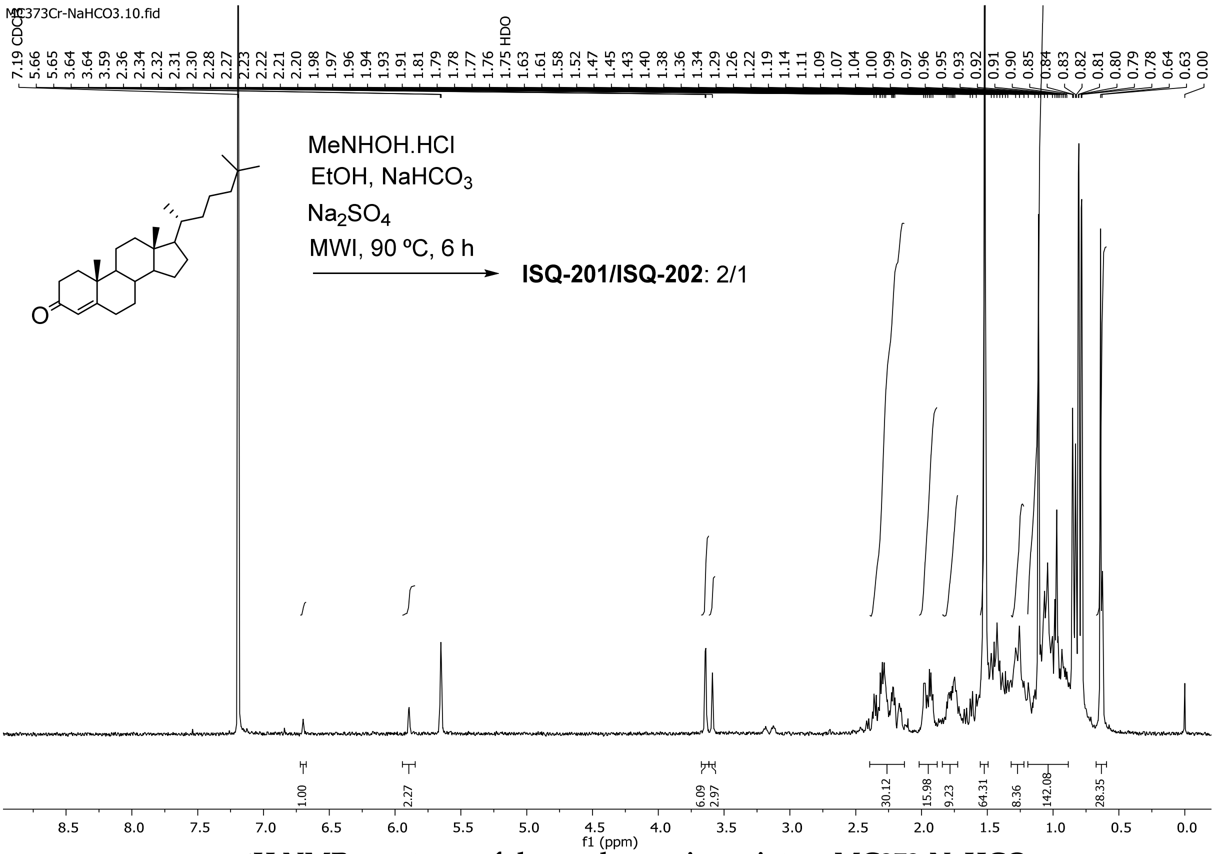

^1H NMR spectrum of the crude reaction mixture MC373-$NaHCO_3$

(C) Following the general procedure, reaction of commercial 4-cholesten-3-one (385 mg, 1 mmol), Na_2SO_4 (426 mg, 3 mmol), **NaOAc** (164 mg, 2 mmol), and methylhydroxylamine hydrochloride (126 mg, 1.5 mmol) in ethanol (10 mL), after 1 h, and column chromatography (CH_2Cl_2/MeOH, from 1% to 3%), gave a mixture of **ISQ-201** and **ISQ-202** in a 3:1 ratio. Repeated and careful chromatography (CH_2Cl_2/MeOH, from 1% to 3%), afforded **ISQ-201** (299 mg, 73%), **ISQ-201 +ISQ-202** (12 mg, 3%), and **ISQ-202** (98 mg, 23%).

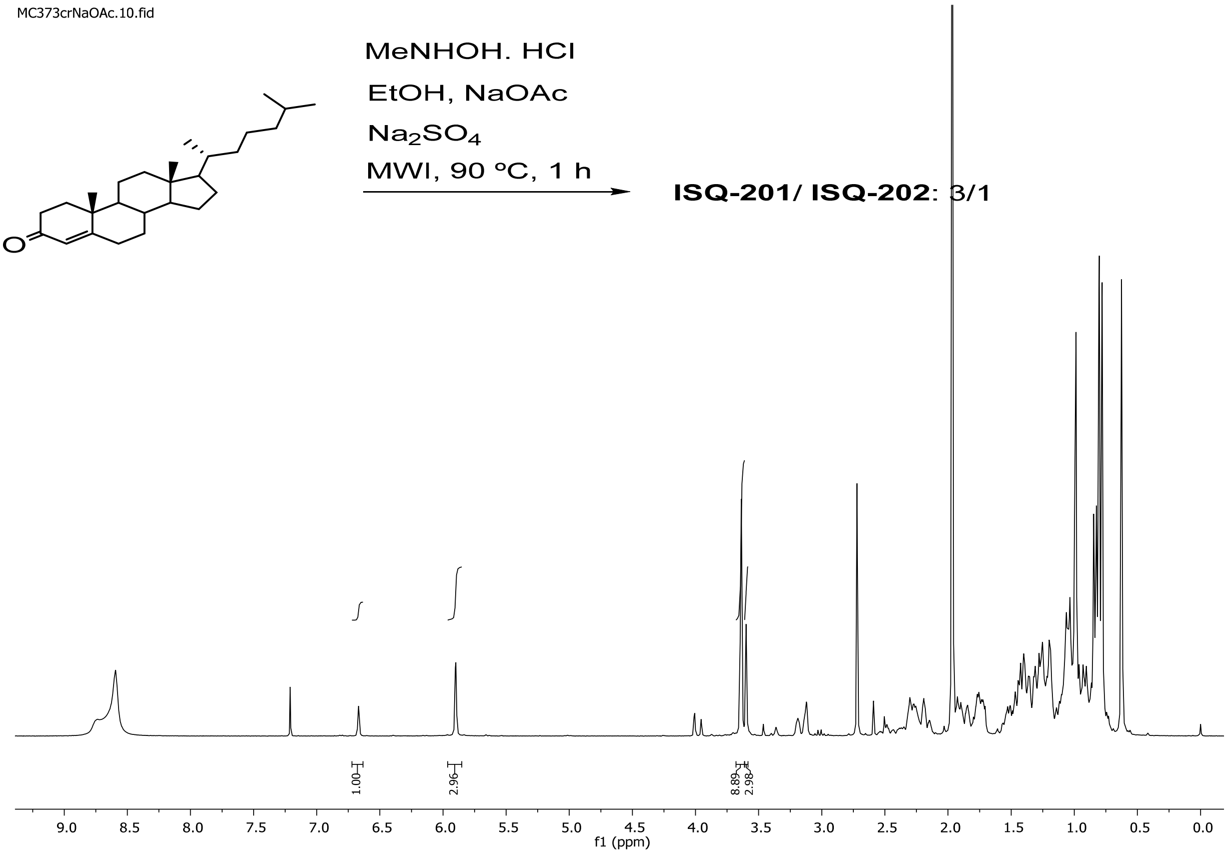

^{1}H NMR spectrum of the crude reaction mixture MC373-AcONa

ISQ-201 : White solid; *Rf* (0.21, CH_2Cl_2/MeOH, 5%); mp 139-141 °C [*anti(= E*) isomer: mp 127-131 ºC[1]]; IR (KBr) ν max 2939, 2868, 2849, 1466, 1215 cm^{-1}; ^{1}H NMR (400 MHz, $CDCl_3$) δ 5.97 (d, *J*= 2.0 Hz, 1H, ^{4}CH), 3.72 (s, 3H, NCH_3), 3.23 (d, *J*= 18.4 Hz, 1H, ^{2}CH), 2.34 (m, 2H, $^{6}CH_2$), 2.21 (m, 1H, ^{2}CH), 1.99 (m, 2H, CH_2), 1.80 (m, 2H, CH_2), 1.60 (s, 3H, CH_3), 1.36 (m, 10H, $5CH_2$), 1.12 (m, 6H, $6CH_2$), 1.04 (s, 3H, $^{19}CH_3$), 0.99 (m, 2H, CH_2), 0.91 (d, *J* = 6.4 Hz, 3H, $^{21}CH_3$), 0.88 (d, *J* = 1.3 Hz, 3H, $^{26}CH_3$), 0.86 (m, 3H, $^{27}CH_3$), 0.70 (s, 3H, $^{18}CH_3$); ^{13}C NMR (101 MHz, $CDCl_3$) δ 156.8 (^{3}C), 146.4 (^{5}C), 112.9 (^{4}CH), 56.1 (^{17}CH), 55.9 (^{14}CH), 53.5 (^{9}CH), 46.0 (^{13}C), 42.3 (NCH_3), 39.6 (C), 39.4 (C), 37.9 (^{10}C), 36.1 (C), 35.77 (C), 35.73 (C), 34.4 (C), 33.4 (C), 32.2 ($^{25}CH_2$), 28.1 ($^{16}CH_2$), 27.9 ($^{2}CH_2$), 24.2 ($^{15}CH_2$), 23.8 ($^{24}CH_2$), 22.7 ($^{26}CH_3$), 22.5 ($^{27}CH_3$), 21.4 (CH_2), 21.3 (^{11}CH), 18.6 ($^{19}CH_3$), 17.8 ($^{21}CH_3$), 11.9 ($^{18}CH_3$). MS (EI) *m/z*: 413 (M, 37%)+, 398 (M-CH_3, 27%), 397(M-O, 70), 137($C_8H_{11}NO$, 100%); MS (ESI) *m/z*: 414.2 (M + H)$^{+}$, 436.2 (M + Na)$^{+}$, 827.8 (2M)$^{+}$, 849.7 (2M + Na)$^{+}$. Anal. Calcd for $C_{28}H_{47}NO$: C, 81.29; H, 11.45; N, 3.39. Found: C, 80.98; H, 11.29; N, 3.44.

ISQ-202: White solid; *Rf* (0.20, CH_2Cl_2/MeOH, 5%); mp 153-155 °C [*syn*(= *E*) isomer: mp 146-149 ºC[1]]; IR (KBr) νmax 2936, 2868, 1629, 1214 cm^{-1}; ^1H NMR (400 MHz, $CDCl_3$) δ ^1H NMR (400 MHz, $CDCl_3$) δ 6.78 (s, 1H, ^4CH), 3.66 (s, 3H, NCH_3), 2.44 (m, 4H, $2CH_2$), 1.88 (m, 4H, $2CH_2$), 1.37 (m, 14H, $7CH_2$), 1.04 (s, 3H, $^{19}CH_3$), 0.98 (m, 2H, CH_2), 0.91 (d, *J*= 6.4 Hz, 3H, $^{21}CH_3$), 0.88 (d, *J* = 1.4 Hz, 3H, $^{26}CH_3$), 0.85 (d, *J* = 1.4 Hz, 3H, $^{27}CH_3$), 0.70 (s, 3H, $^{18}CH_3$); ^{13}C NMR (101 MHz, $CDCl_3$) δ 123.7 (^3C), 120.3 (^5C), 113.7 (^4CH), 56.0 (^{17}CH), 55.9 (^{14}CH), 53.5 (^9CH), 46.4 (^{13}C), 42.3 (NCH_3), 39.6 (C), 39.4 (C), 37.9 (^{10}C), 36.0 (C), 35.72 (C), 35.71 (C), 35.4 (C), 32.9 (C), 32.2 (C), 28.1 (C), 27.9 (C), 24.1 ($^{16}CH_2$), 23.7 ($^{15}CH_2$), 23.6 ($^{24}CH_2$), 22.7 ($^{26}CH_3$), 22.5 ($^{27}CH_3$), 21.3 ($^{11}CH_2$), 18.6 ($^{19}CH_3$), 17.8 ($^{21}CH_3$), 11.9 ($^{18}CH_3$). MS (EI) *m/z:* 413 (M, 37%)$^+$, 398 (M-CH_3, 27%), 397(M-O, 70), 137 ($C_8H_{11}NO$, 100%); MS (ESI) *m/z*: 414.2 (M + H)$^+$, 827.8 (2M)$^+$, 849.7 (2M + Na)$^+$. Anal. Calcd for $C_{28}H_{47}NO$: C, 81.29; H, 11.45; N, 3.39. Found: C, 81.03; H, 11.33; N, 3.30.

References

(1) Barton, D. H. R.; Day, M. J.; Hesse, R. H.; Pechet, M. M. A new rearrangement of ketonic nitrones; a convenient alternative to the Beckmann rearrangement. *J. Chem. Soc., Perkin Trans. 1* **1975**, 1764-1767.

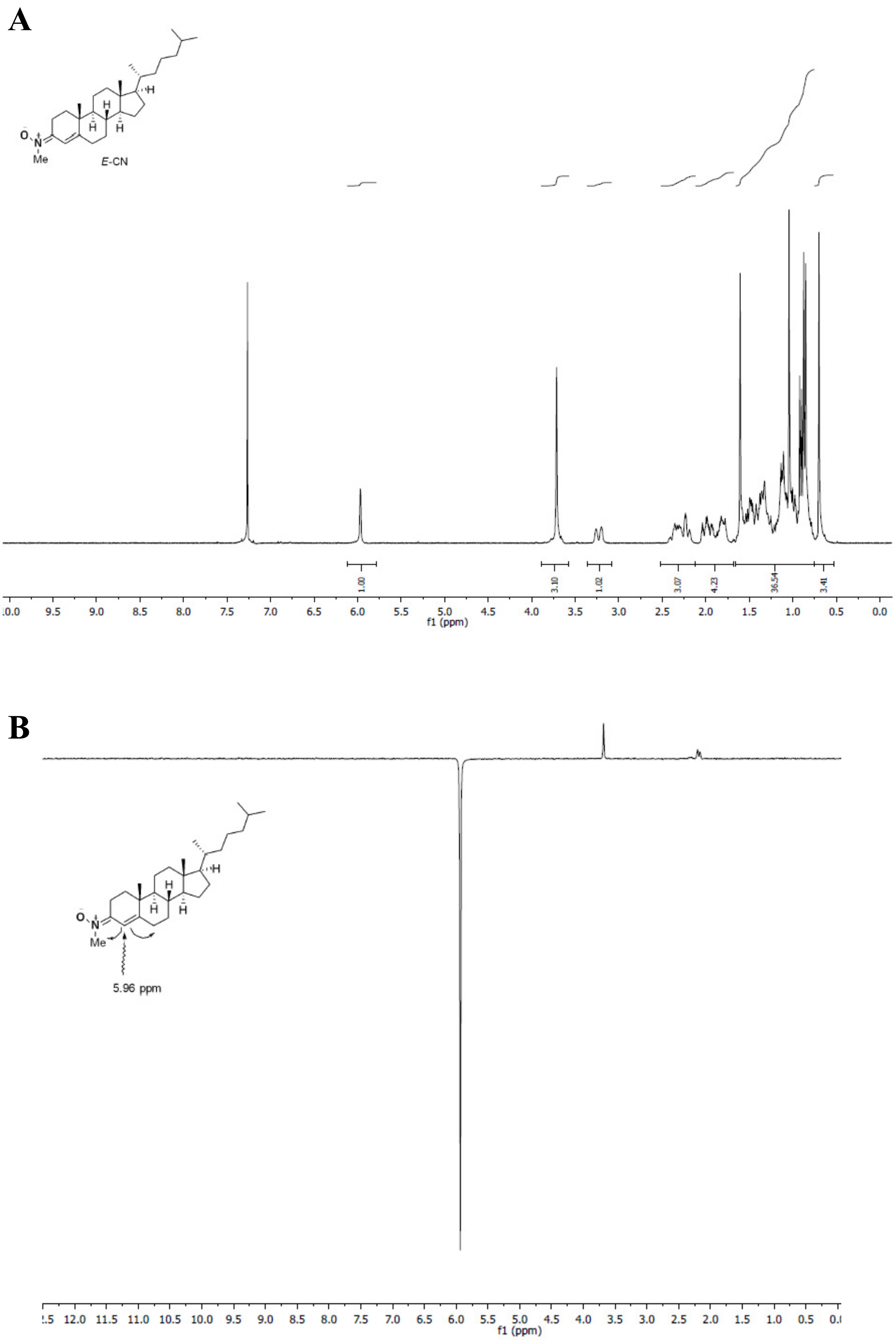

Figure 2. (**A**) ^{1}H NMR spectrum of ChN **ISQ-201** . (**B**) Selective ^{1}H NMR nOe spectrum of ChN **ISQ-201** .

A

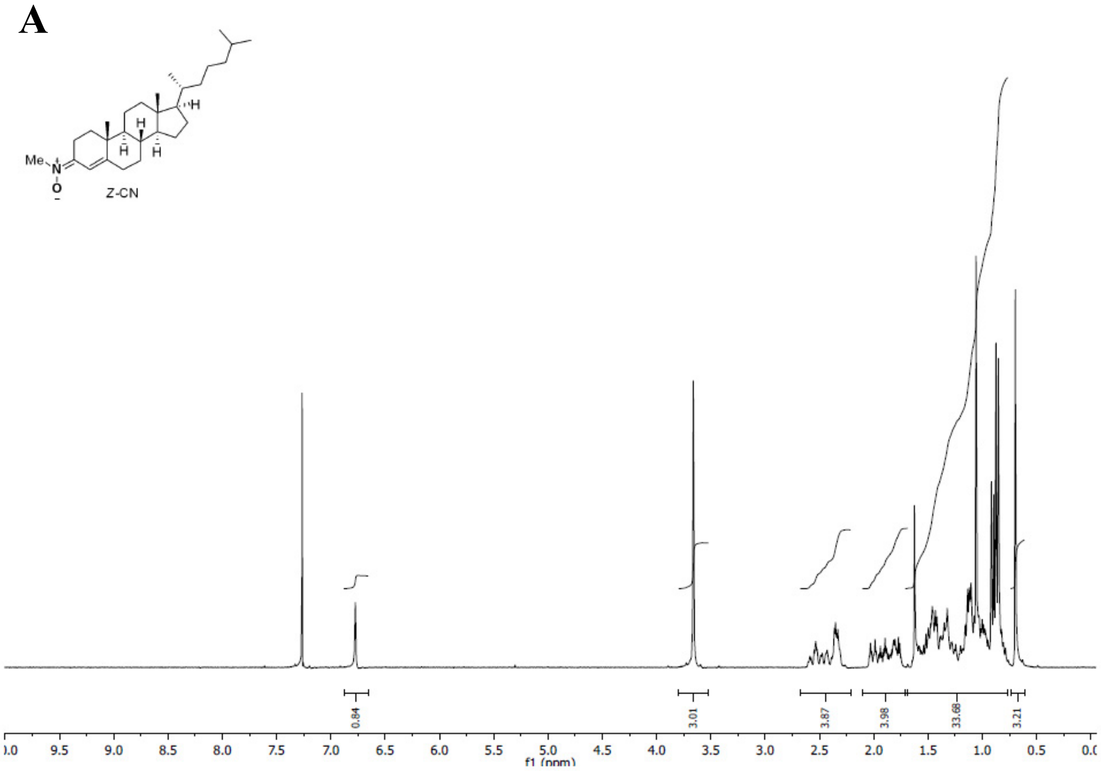

B

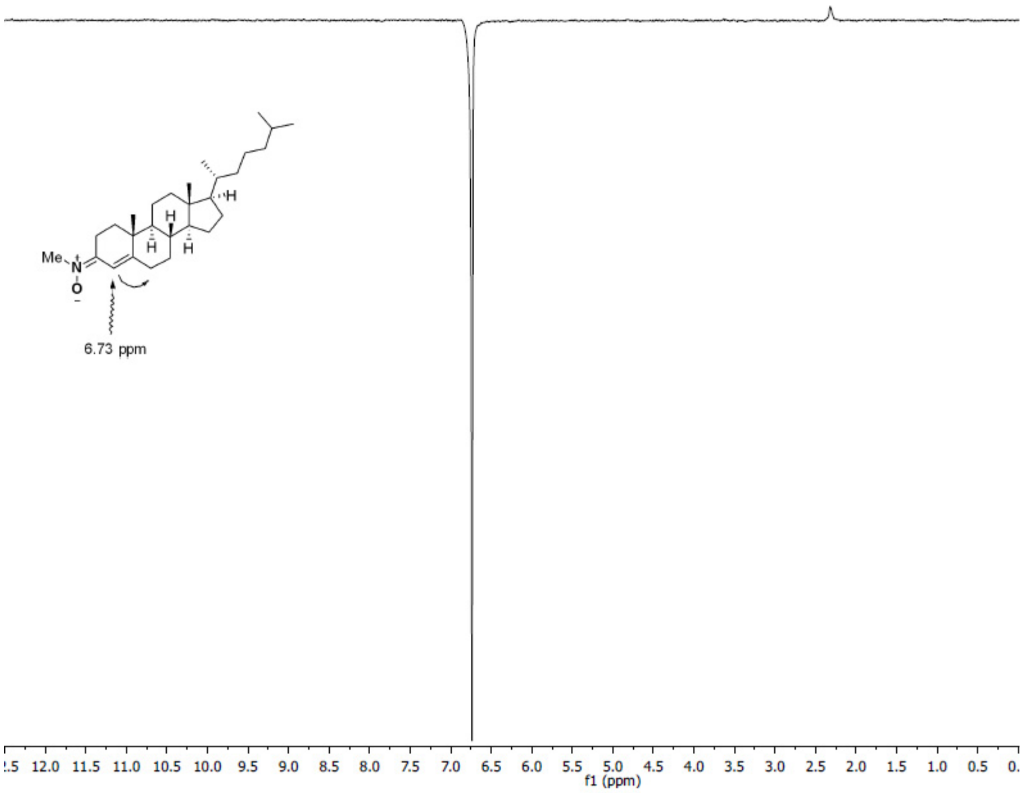

Figure 3. (A) ¹H NMR spectrum of ChN **ISQ-202**. (**B**) Selective ¹H NMR nOe spectrum of ChN **ISQ-202**.

B. Synthesis of cholesteronitrone ISQ-201 from natural cholesterol-plant

1. Oxidation of cholesterol

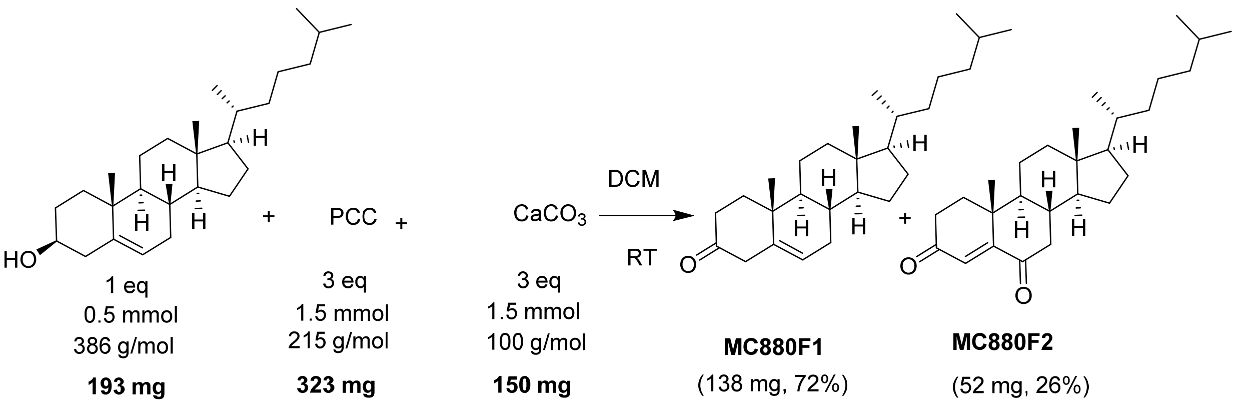

Following the described method (A. K. Batta, G. Saien, S. Shefer *Steroids* **1988**, *52*, 109-122), to a suspension of PCC (323 mg, 1.5 mmol, 3 equiv), $CaCO_3$ (150 mg, 1.5 mmol, 3 equiv) en DCM (10 mL), a solution of cholesterol-plant (193 mg, 0.5 mmol) in DCM (5 mL) was added. Then, the mixture was stirred at rt for 45 min, was filtered over Celite, washed with DCM, the solvent was evaporated, and the residue was recrystallized from methanol, affording a solid that was purified by column chromatography, using hexane/AcOEt (from 1% to 10%), to give: cholest-5-en-3-ona (**MC880F1**, 138 mg, 72%), and cholest-4-en-3,6-dione (**MC880ISQ-201** , 52 mg (26%) (J.-G. Cui, L. Fan, L.-L. Huang, H.-L. Liu, A.-M. Zhou, *Steroids* **2009**. *74*, 62-72).

¹H NMR MC880F1

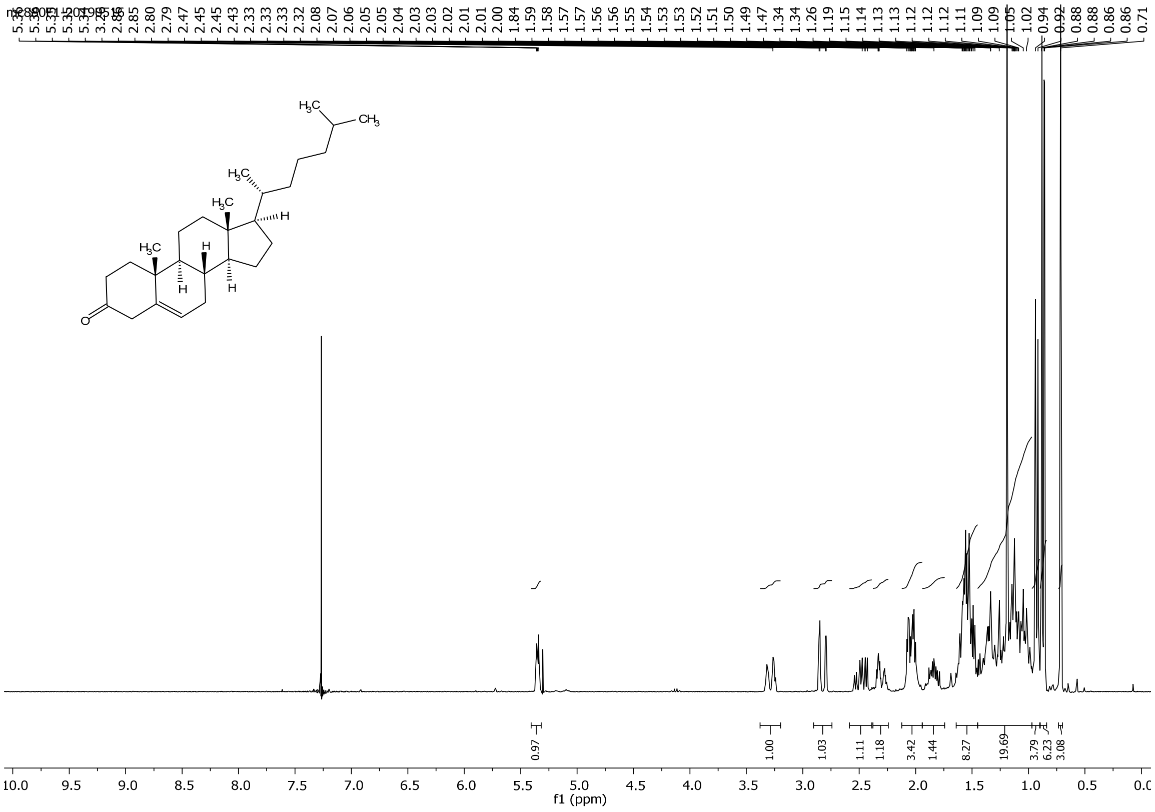

¹H NMR- MC880ISQ-201

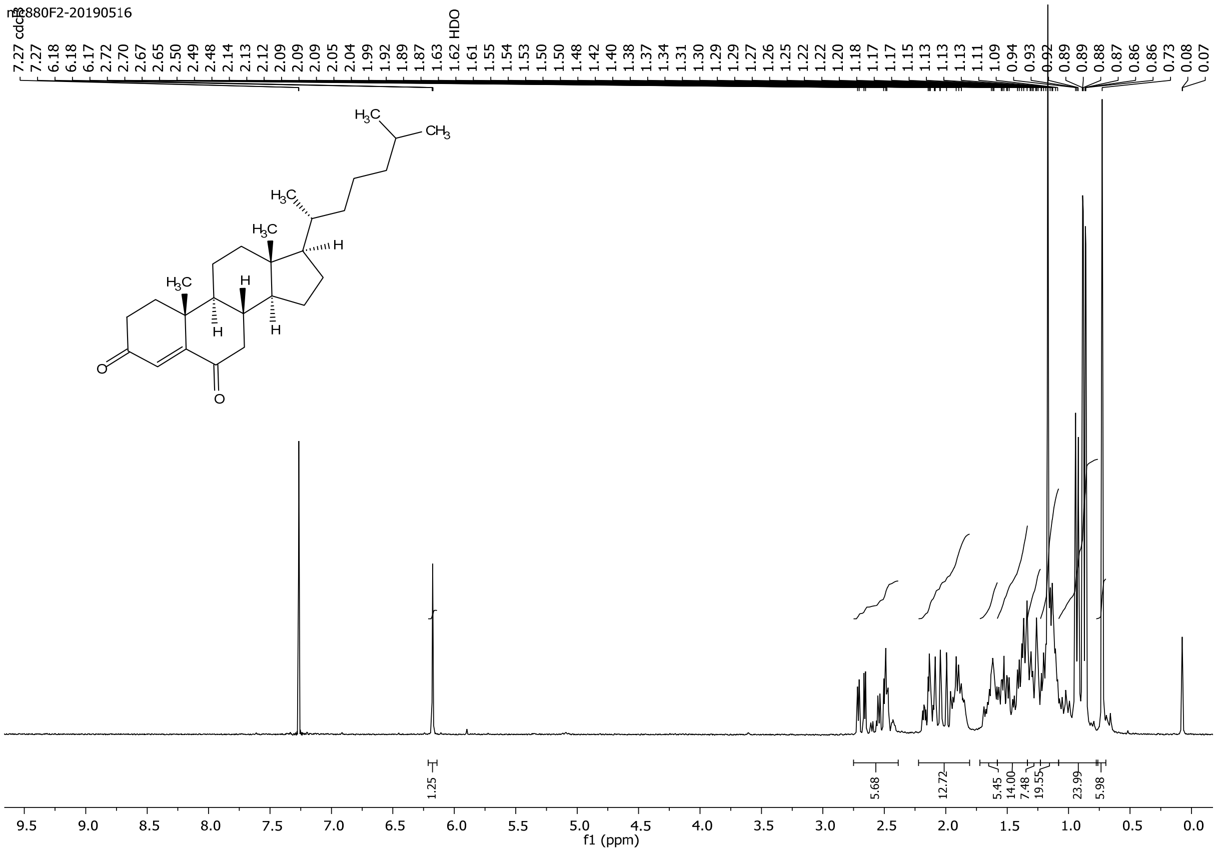

2. Isomerization of compound MC880F1

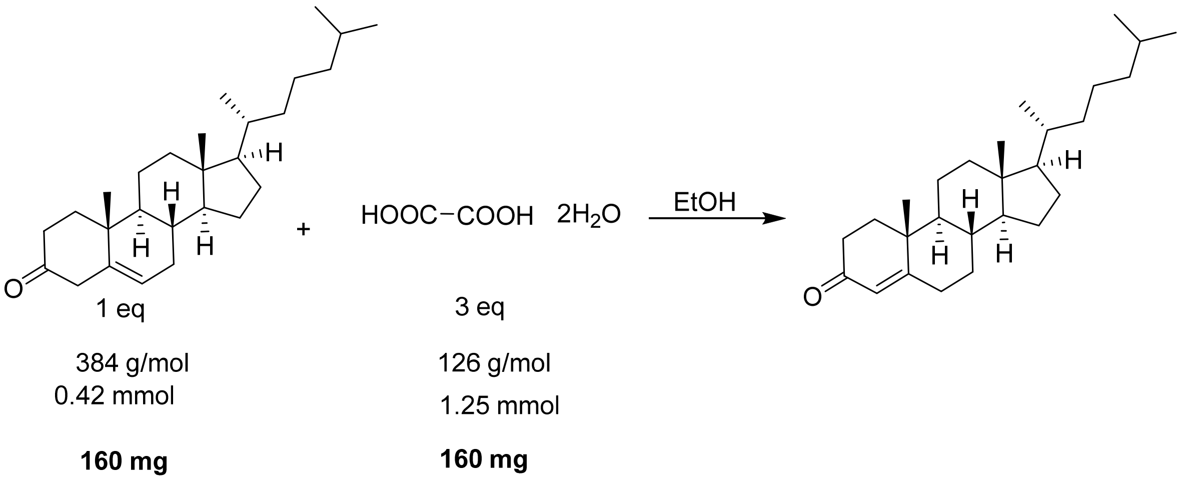

Following the described method (J.-G. Cui, L. Fan, L.-L. Huang, H.-L. Liu, A.-M. Zhou *Steroids* **2009**. *74*, 62-72), a solution of **MC880F1** (160 mg, 0.42 mmol) in EtOH (5 mL) was treated with oxalic acid monohydrate (160 mg, 1.25 mmol, 3 equiv), at reflux for 24 h. The mixture was cooled, the solvent evaporated, and the residue was dissolved in DCM, washed with water, and extracted several (x 3) times. The organic phase was dried with Na_2SO_4, filtered, and the solvent was evaporated to give pure **MC881F1** (155 mg, 97%).

^{1}H NMR- MC881F1

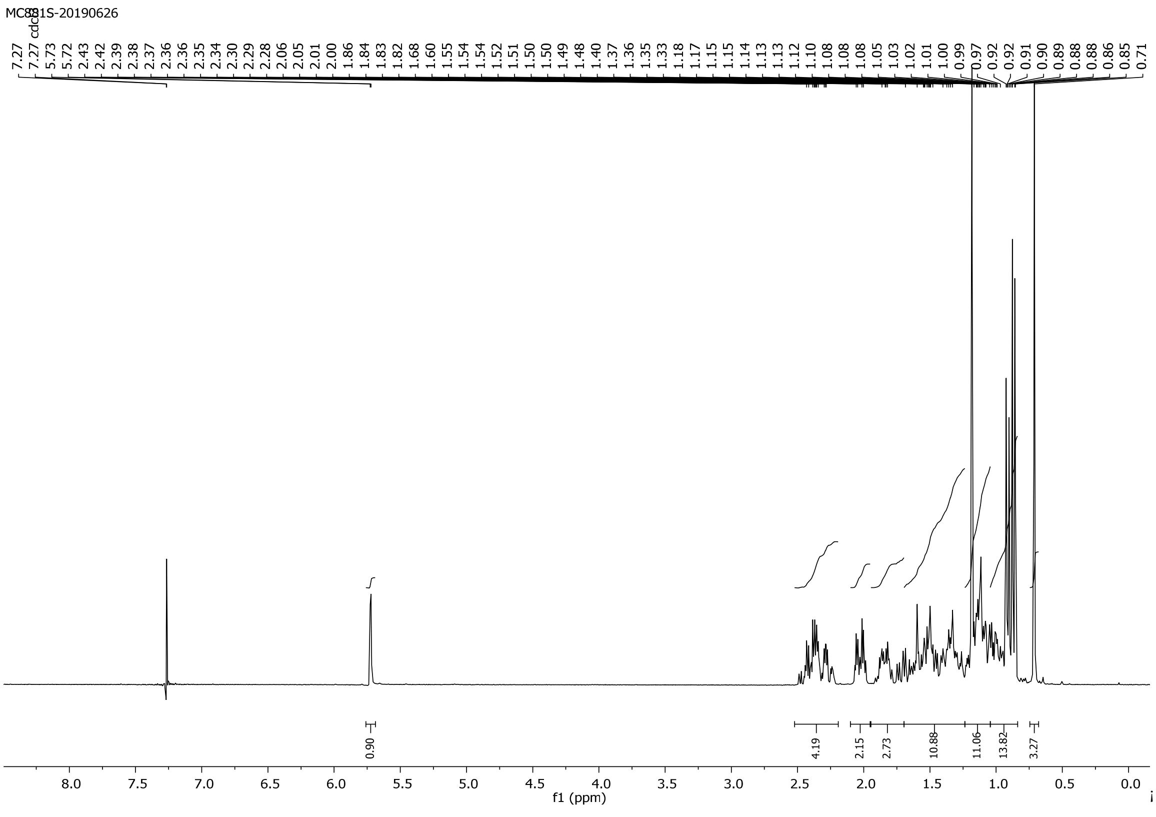

3. Synthesis of ISQ-201 plant

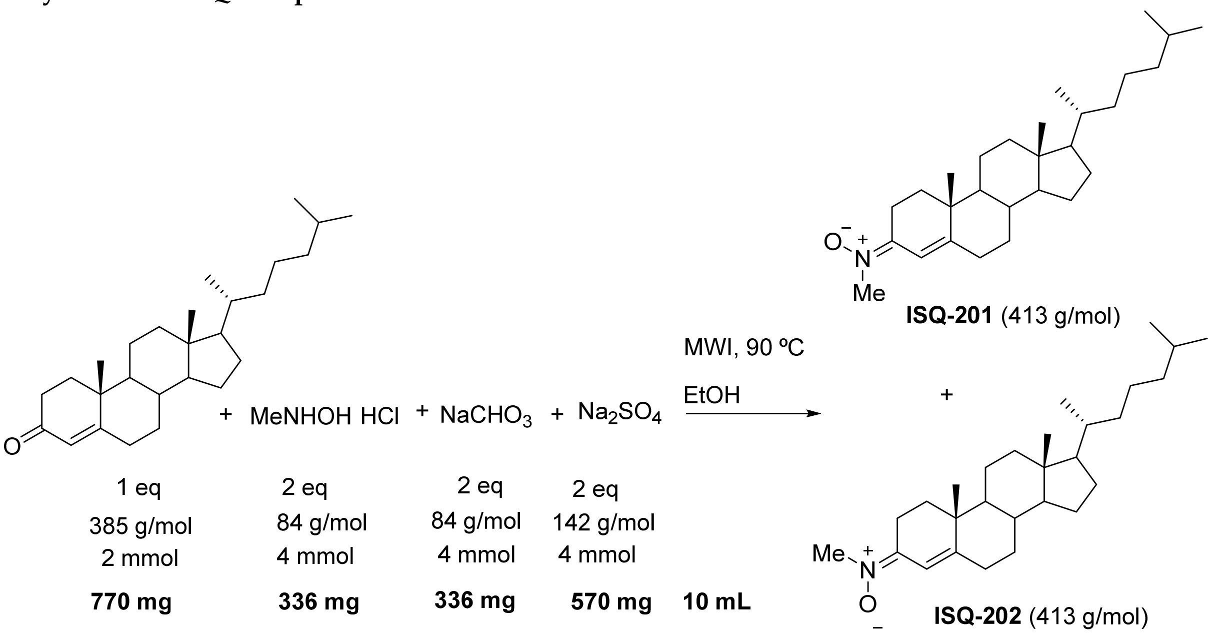

A suspension of cholest-4-en-3-one (**MC881F1**) (770 mg, 2 mmol), *N*-methyl hydroxylamine. HCl (336 mg, 4 mmol, 2 equiv), $NaHCO_3$ (336 mg, 2 mmol), and Na_2SO_4 (570 mg, 4 mmol) in ethanol (10 mL), was irradiated (250 W) for 3 h at 90 ºC. After cooling the mixture, the solvent was removed, and the residue was purified by column chromatography, using DCM/methanol (from 1% to 3%) to give **ISQ-201** (410 mg, 49%), **ISQ-201** + **2ISQ-202** (25 mg, 3%) and **ISQ-202** (205 mg, 25%).

ISQ-201 : White solid; *Rf* (0.21, CH_2Cl_2/MeOH, 5%); mp 139-141 °C [*anti*(= *E*) isomer: mp 127-131 ºC[1]]; IR (KBr) ν max 2939, 2868, 2849, 1466, 1215 cm^{-1}; ^{1}H NMR (400 MHz, $CDCl_3$) δ 5.97 (d, *J*= 2.0 Hz, 1H, ^{4}CH), 3.72 (s, 3H, NCH_3), 3.23 (d, *J*= 18.4 Hz, 1H, ^{2}CH), 2.34 (m, 2H, $^{6}CH_2$), 2.21 (m, 1H, ^{2}CH), 1.99 (m, 2H, CH_2), 1.80 (m, 2H, CH_2), 1.60 (s, 3H, CH_3), 1.36 (m, 10H, $5CH_2$), 1.12 (m, 6H, $6CH_2$), 1.04 (s, 3H, $^{19}CH_3$), 0.99 (m, 2H, CH_2), 0.91 (d, *J* = 6.4 Hz, 3H, $^{21}CH_3$), 0.88 (d, *J* = 1.3 Hz, 3H, $^{26}CH_3$), 0.86 (m, 3H, $^{27}CH_3$), 0.70 (s, 3H, $^{18}CH_3$); ^{13}C NMR (101 MHz, $CDCl_3$) δ 156.8 (^{3}C), 146.4 (^{5}C), 112.9 (^{4}CH), 56.1 (^{17}CH), 55.9 (^{14}CH), 53.5 (^{9}CH), 46.0 (^{13}C), 42.3 (NCH_3), 39.6 (C), 39.4 (C), 37.9 (^{10}C), 36.1 (C), 35.77 (C), 35.73 (C), 34.4 (C), 33.4 (C), 32.2 ($^{25}CH_2$), 28.1 ($^{16}CH_2$), 27.9 ($^{2}CH_2$), 24.2 ($^{15}CH_2$), 23.8 ($^{24}CH_2$), 22.7 ($^{26}CH_3$), 22.5 ($^{27}CH_3$), 21.4 (CH_2), 21.3 (^{11}CH), 18.6 ($^{19}CH_3$), 17.8 ($^{21}CH_3$), 11.9 ($^{18}CH_3$). MS (EI) *m*/*z*: 413 (M, 37%)+, 398 (M-CH_3, 27%), 397(M-O,

70), 137($C_8H_{11}NO$, 100%); MS (ESI) *m/z*: 414.2 (M + H)$^+$, 436.2 (M + Na)$^+$, 827.8 (2M)$^+$, 849.7 (2M + Na)$^+$. Anal. Calcd for $C_{28}H_{47}NO$: C, 81.29; H, 11.45; N, 3.39. Found: C, 80.98; H, 11.29; N, 3.44.

ISQ-202: White solid; *Rf* (0.20, CH_2Cl_2/MeOH, 5%); mp 153-155 °C [*syn*(= *E*) isomer: mp 146-149 ºC[1]]; IR (KBr) νmax 2936, 2868, 1629, 1214 cm^{-1}; ^{1}H NMR (400 MHz, $CDCl_3$) δ ^{1}H NMR (400 MHz, $CDCl_3$) δ 6.78 (s, 1H, ^{4}CH), 3.66 (s, 3H, NCH_3), 2.44 (m, 4H, $2CH_2$), 1.88 (m, 4H, $2CH_2$), 1.37 (m, 14H, $7CH_2$), 1.04 (s, 3H, $^{19}CH_3$), 0.98 (m, 2H, CH_2), 0.91 (d, *J*= 6.4 Hz, 3H, $^{21}CH_3$), 0.88 (d, *J* = 1.4 Hz, 3H, $^{26}CH_3$), 0.85 (d, *J* = 1.4 Hz, 3H, $^{27}CH_3$), 0.70 (s, 3H, $^{18}CH_3$); ^{13}C NMR (101 MHz, $CDCl_3$) δ 123.7 (^{3}C), 120.3 (^{5}C), 113.7 (^{4}CH), 56.0 (^{17}CH), 55.9 (^{14}CH), 53.5 (^{9}CH), 46.4 (^{13}C), 42.3 (NCH_3), 39.6 (C), 39.4 (C), 37.9 (^{10}C), 36.0 (C), 35.72 (C), 35.71 (C), 35.4 (C), 32.9 (C), 32.2 (C), 28.1 (C), 27.9 (C), 24.1 ($^{16}CH_2$), 23.7 ($^{15}CH_2$), 23.6 ($^{24}CH_2$), 22.7 ($^{26}CH_3$), 22.5 ($^{27}CH_3$), 21.3 ($^{11}CH_2$), 18.6 ($^{19}CH_3$), 17.8 ($^{21}CH_3$), 11.9 ($^{18}CH_3$). MS (EI) *m/z:* 413 (M, 37%)$^+$, 398 (M-CH_3, 27%), 397(M-O, 70), 137 ($C_8H_{11}NO$, 100%); MS (ESI) *m/z*: 414.2 (M + H)$^+$, 827.8 (2M)$^+$, 849.7 (2M + Na)$^+$. Anal. Calcd for $C_{28}H_{47}NO$: C, 81.29; H, 11.45; N, 3.39. Found: C, 81.03; H, 11.33; N, 3.30.

^{1}H NMR ISQ-201

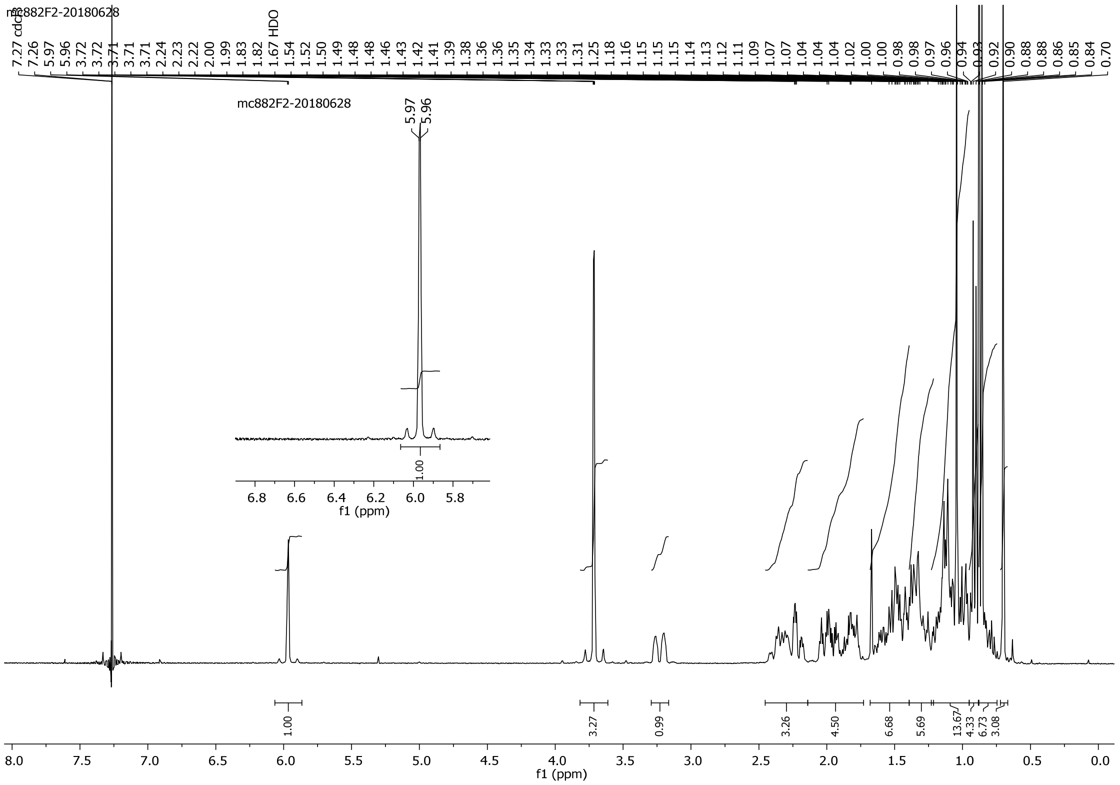

^{1}H NMR ISQ-202

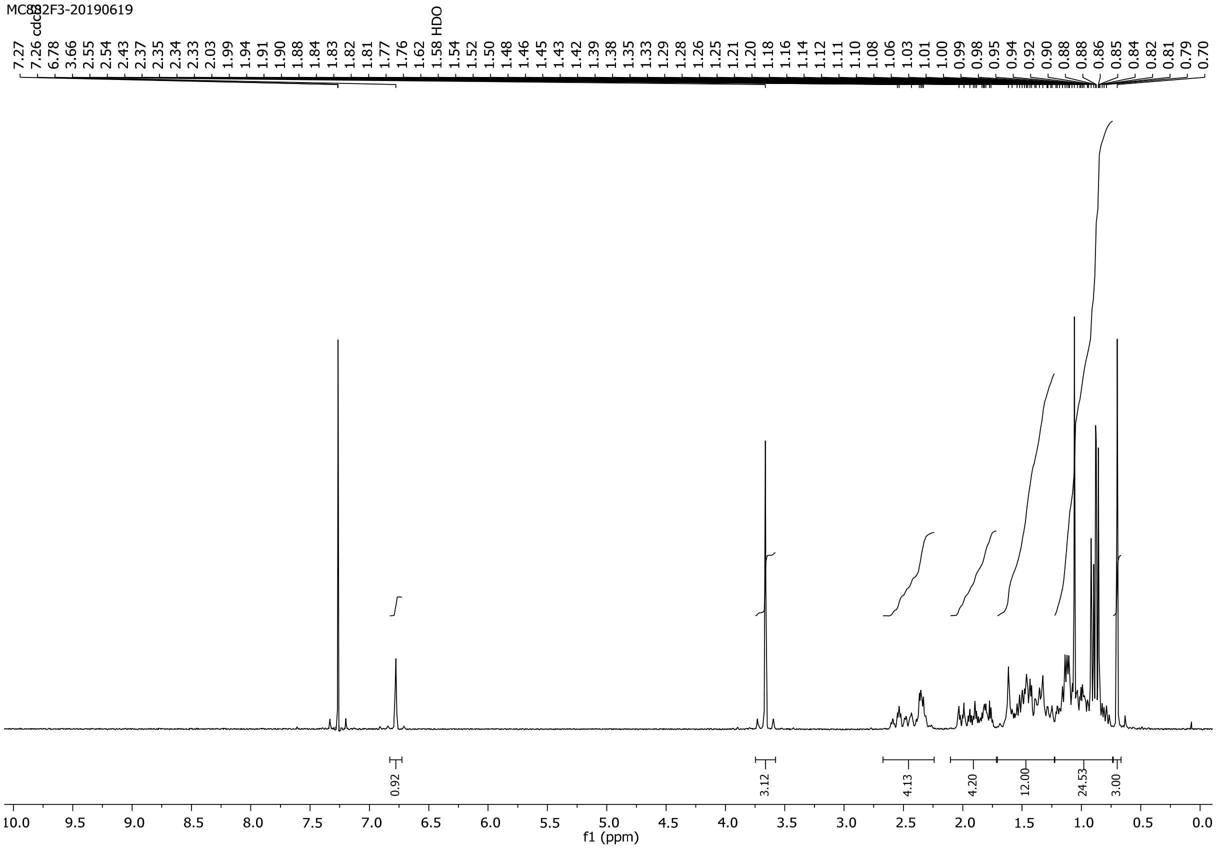

Supplement: Supplementary file 1 [file antioxidants-09-00291-s001.pdf]
